# Antioxidant processes involving epicatechin decreased symptoms of pine wilt disease

**DOI:** 10.3389/fpls.2022.1015970

**Published:** 2022-12-09

**Authors:** Ruizhi Zhang, Jianan Wang, Rui Xia, Danlei Li, Feng Wang

**Affiliations:** ^1^ Key Laboratory of Alien Forest Pest Detection and Control-Heilongjiang Province, School of Forestry, Northeast Forestry University, Harbin, China; ^2^ Liaoning Provincial Key Laboratory of Dangerous Forest Pest Management and Control, Liaoning forestry and grassland Bureau, Fushun, China

**Keywords:** *Bursaphelenchus xylophilus*, transcriptome, metabolome, antioxidant, epicatechin, H_2_O_2_

## Abstract

Since the pine wood nematode (PWN, *Bursaphelenchus xylophilus*) invasion of Northeast China, both symptomatic and asymptomatic PWN carriers have been found. Asymptomatic PWN carriers, which are more dangerous than symptomatic carriers, constitute a source of infection in the following spring. The simultaneous presence of symptomatic and asymptomatic PWN carriers indicates that *Pinus koraiensis* has different tolerance levels to PWN. In this study, validity of susceptibility testing discovered differential types of *P. koraiensis* including Latent Reservoirs, Low Susceptibles, High Susceptibles and Bell Ringers. Among those types, the Low Susceptibles and Latent Reservoirs were asymptomatic PWN carriers, and Latent Reservoirs were the most dangerous. Transcriptome and metabolomic data showed that 5 genes (3 *ans* and 2 *anr* gene) involved in the epicatechin (EC) synthesis pathway were significantly upregulated, which increased the content of EC antioxidants in Latent Reservoirs. Hydrogen peroxide (H_2_O_2_) staining and content determination showed that the hypersensitive response (HR) and H_2_O_2_, which functions as a signaling molecule in systemic acquired resistance, decreased in Latent Reservoirs. However, low contents of EC and high contents of H_2_O_2_ were found in the High Susceptibles of *P. koraiensis*. RT-PCR results showed that the expression of *ans* and *anr* was upregulated together only in Latent Reservoirs. These results show that the susceptibility of *P. koraiensis* to PWN differed among different individuals, although no resistant individuals were found. Latent Reservoirs, in which more PWNs resided without visible symptoms *via* prolonged incubation period, inhibited the symptoms caused by H_2_O_2_ because of increased contents of the EC antioxidants.

## Introduction

As an obligate endoparasitic nematode of Pinaceae species, pine wood nematode (PWN, *Bursaphelenchus xylophilus*) causes pine wilt disease (PWD) and major ecological damage (Futai, 2013). Although native to North America, PWN has caused more severe damage to pine trees in East Asia, especially in China (Ye, 2019). Recently, PWNs have crossed the line where the annual average temperature is 10°C and expanded to Liaoning and Jilin in Northeast China (Zhao et al., 2017; Li and Zhang, 2018; Hou et al., 2021; Li et al., 2022a). With the entry of PWN into Liaoning, it was found that *Pinus koraiensis* was also a natural host (Yu and Wu, 2018). *P. koraiensis*-broadleaf mixed forests are typical zonality forests in the northeastern region of China. Moreover, asymptomatic PWN carriers were found in *P. koraiensis* after PWN infection in Liaoning Province. Identifying incipient symptoms of asymptomatic PWN carriers is difficult (Futai, 2013) until the symptoms are visible in the next year. Asymptomatic carriers are the leading cause of the rapid spread of PWN.

To determine the pathogenesis of pine trees infected with PWN, many physiological (Futai, 2013; Kenichi et al., 2018), biochemical (Yamada, 2006; Yamada, 2008), histological and structural (Futai, 2013) studies have been performed. Although the enzyme hypothesis, the toxin hypothesis and the cavitation hypothesis have been proposed for the pathogenesis of PWD (Yang et al., 2003), the infection processes of host plants are unclear. In a previous study, it was found that pine trees showed a hypersensitive response (HR) after PWN infection (Mamiya, 1983; Jones and Dangl, 2006). Beginning with an oxidative burst of reactive oxygen species (ROS) (Lamb and Dixon, 1997), the HR is activated to limit the growth of obligatory parasitic pathogens (Heath, 2000; Nanda et al., 2010). Consequently, a large number of ROS accumulate at the infection site of the pathogen (Apel and Hirt, 2004; Gill and Tuteja, 2010). High levels of ROS production are lethal to cellular integrity. However, high levels of ROS production are also necessary for plant defence (Nanda et al., 2010). ROS accumulation is the key to plant disease resistance. In response to oxidative stress, plants have developed antioxidant defense systems that involve enzymatic and nonenzymatic antioxidant reactions to maintain normal cellular metabolism and function to protect themselves from oxidative stress (Gill and Tuteja, 2010; Ren et al., 2021). Suppression of ROS toxicity and control of ROS accumulation in plants requires a large network of genes (Mittler et al., 2004). Elucidating this process is helpful to better understand the pathological process of PWD.

Epicatechin (EC) is flavonoid with strong antioxidant activity (Xu et al., 2004). EC has direct antioxidant activity, but this activity is limited to tissues that contain high amounts of EC (Fraga et al., 2018). Proanthocyanidins (PAs, including catechin and EC) are an antioxidant that scavenge harmful free radicals in cells and have a very high antioxidant capacity, 20 times that of vitamin C and 50 times that of vitamin E (Shi et al., 2003; Xin et al., 2020). The biological activity of EC is mainly attributed to their interaction with proteins and lipids, which can affect the level of oxidants in cells (Fraga et al., 2018). PAs are essential for the antioxidant activity of fruits and are also prefabricated or induced antibacterial agents in plants (Goupil et al., 2020; Xin et al., 2020). EC is an important inducible defensive compound in tea plant (Li et al., 2022b). In the mulberry, the content of EC in transgenic mulberry was increased by *MnANR* transgenic, and the resistance to *Botrytis cinerea* was improved (Xin et al., 2020). EC has been reported to inhibit the appressorial melanization of the necrotrophic fungus *Colletotrichum kahawae* causing coffee-berry disease (Chen et al., 2006). Epicatechin is also an effective chemical to prevent rust infection (Ullah et al., 2017). The above studies show that EC is critical to plant disease resistance. The biosynthesis of EC involves the conversion of leucoanthocyanidins into anthocyanins by anthocyanin synthase (ANS) followed by the reduction of anthocyanins to corresponding 2,3-cis-flavan-3-alcohol, which is EC, by anthocyanin reductase (ANR) (Xu et al., 2012). When *ans* is introduced into plants and overexpressed, the accumulation of anthocyanin glycosides in plants changes, and the antioxidant capacity of plants increases (Liu et al., 2021). Moreover, overexpression of *anr* can increase the level of PAs and improve the antioxidant capacity of plants (Wang et al., 2013).

Metabolic profiling by integrating the metabolome with other omics tools has proven to be very effective in functional gene identification and pathway elucidation in plant pro-metabolism and secondary metabolism (Zhu et al., 2018). In this study, 4 different types of *P. koraiensis* were discovered, which were selected on the basis of their symptoms of PWD and the number of isolated PWNs. Transcriptome sequencing was used to compare the differentially expressed genes between Latent Reservoirs and the other types, and antioxidant-related genes were identified. The contents of PWN-related hydrogen peroxide (H_2_O_2_) and EC in the different *P. koraiensis* types were determined by UV spectroscopy and metabolomics. The oxidation and antioxidation mechanisms of host plants infected with PWN were investigated to elucidate the pathogenesis of PWD.

## Materials and methods

### Biological material

**Figure 1:**
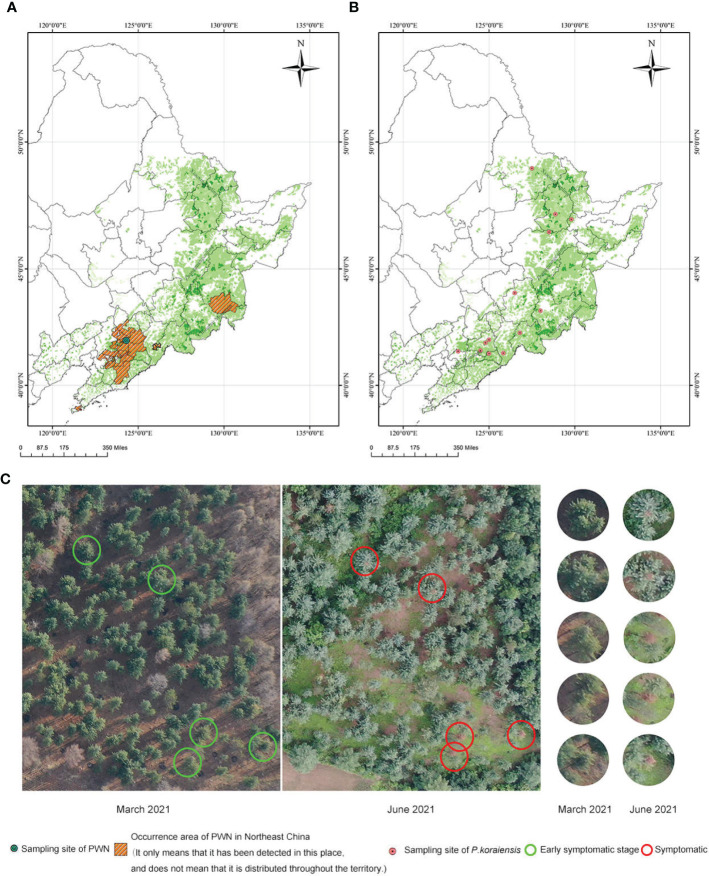
Distribution of the collected samples and unmanned aerial vehicle-collected images of the PWN sampling site. **(A)** Occurrence and sampling sites of PWN in Northeast China (Data source: up to March 2022). The basemap is the color English version of the map of China (including POI) in ArcGIS. **(B)** *P. koraiensis* sampling sites in the experiment **(C)** Over-year death of *P. koraiensis* in the PWN sampling area (In 2020, *P. koraiensis* trees in this area were infected with PWN through *M. saltuarius*. In March 2021, some *P. koraiensis* trees in the area showed initial symptoms. In June 2021, *P. koraiensis* trees that were infected by PWN were completely symptomatic.).

The FS*Bx* population of PWN used in the experiment was collected from *P. koraiensis* infected with PWN in Fushun city, Liaoning Province (**Figure 1A**). FS*Bx* were cultured on *B. cinerea* fungi at 25°C in the dark for propagation. All *in vivo* PWN tests were completed in Liaoning under controlled laboratory condition. After the test, all materials carry PWN were sent to sterilization for conducting harmless treatment.

Sixty *P. koraiensis* individuals were collected from 13 seed orchards in a nonepidemic area of Northeast China (**Figure 1B**). The two-year-old branches of each *P. koraiensis* individual were cut and used as experimental materials.

### Plant inoculation and symptom evaluation

Cultured nematodes (a mixture of adult and juvenile nematodes in a male to female to juvenile ratio of approx. 1:1:2) were added to ddH_2_O to adjust the concentration of the PWN suspension to 50 strips per microliter. The 60 *P. koraiensis* individuals were inoculated following the method (Wang et al., 2019). Healthy two-year-old branches of each *P. koraiensis* individual were removed, cleaned, and placed in a 100 mL conical flask. Fifty milliliters of ddH_2_O were added to each 100 mL conical flask. These two-year-old branches were cultivated in a greenhouse in Fushun City, Liaoning Province (16 h of light, 8 h of darkness), at 25°C for 1 day. Several needles in the middle of the two-year-old branches were removed to expose a small stem segment. A sterile scalpel was used to cut a small longitudinal wound at the exposed stem segment. In the treatment group, a suspension with 5,000 FS*Bx* (100 μL PWN suspension) was pipetted into the small longitudinal wound; in the CK group, 100 μL of ddH_2_O was pipetted into the small longitudinal wound. The inoculated wounds were covered with parafilm to prevent drying of the inocula.

All 60 *P. koraiensis* individuals were imaged, and the disease infection index of each individual was recorded every day (Modesto et al., 2021). Plants for which less than 1/4 of whose needles were partially chlorotic were classified as disease grade I. Plants for which half of whose needles were chlorotic, with the tips curved, were classified as disease grade II. Plants whose majority of needles were chlorotic, yellowing and browning, with shoots bent and drooping, were classified as disease grade III. Plants of whose all needles were brown and whole plants that were dead were classified as disease grade IV.

When the disease infection index of one *P. koraiensis* individual reached grade IV, the symptoms of 60 *P. koraiensis* individuals were imaged and recorded. The ratio of green and red needles of *P. koraiensis* in the images was calculated according to color clusters, which were calculated using *k*-means clustering. The disease infection index of each *P. koraiensis* individual was calculated from the ratio of green to red needles. The calculation formula was as follows:


$$disease\;infection\;index=1-\frac{proportion\;of\;green\;needles}{proportion\;of\;all\;needles}$$

The *P. koraiensis* trees in the treatment group and CK group of 60 individuals were sliced by hand at 33 days post-inoculation (dpi). The transverse sections were made in segments 1 cm above the inoculation sites. The trees in the treatment group and CK group were cut such that 6 transverse sections were obtained from each individual.

### Isolation of PWN

The PWNs were isolated from the branches of the 60 *P. koraiensis* individuals by the Behrman funnel method (Chen et al., 2021b). The isolated PWNs of each *P. koraiensis* individual were imaged and counted *via* a dissecting microscope. Then, the PWNs were stained with green fluorescent dye. The stained PWNs were imaged under a 492 nm excitation wavelength by a fluorescence microscope (OLYMPUS, BX51).

### RNA extraction, library construction, and sequencing

Total RNA from the treatment and CK groups with 4 different types of *P. koraiensis* trees at 12 h after treatment was extracted by cetyl-trimethylammonium bromide (CTAB) and then cleaned through an Rneasy Mini Kit column (Qiagen, Valencia, CA, USA) (Chen et al., 2021b). There were 3 biological replicates in the treatment and CK groups of 4 different types of *P. koraiensis*. The purity and integrity of the extracted RNA were evaluated by agarose gel electrophoresis and a NanoDrop microvolume spectrophotometer (Thermo Scientific, Wilmington, DE, USA). Transcriptome sequencing was performed on an Illumina HiSeq 150 platform (BGI, Shenzhen, China). FastQC was used to evaluate the quality of the sequencing data. RNA libraries were constructed using Qubit and an Agilent 2100 instrument. The original data was further filtered to obtain high-quality data. The filtering standards included removing the adapter sequence of version 2 by the adapter, quality filtering and length filtering by the sliding window method (removing sequences less than 50 bp).

### Identification of differentially expressed genes

The read distribution of gene expression levels in each library was normalized to construct an effective library size. The differentially expressed *P. koraiensis* genes were analyzed according to the method of Li et al. (Li et al., 2016). The expression levels of the differentially expressed genes were compared using the log_2_(treatment/CK fold-change) of the normalized read levels. Multiple hypothesis test corrections were performed according to the *P* values of the tests, and the domain value of the *P* value was determined by controlling the false discovery rate (FDR) (Benjamini and Yekutieli, 2001). The threshold of differentially expressed genes was set to log_2_(treatment/CK fold-change) >  1 or  <  −1 (FDR < 0.05). Similarly, the threshold for significant differentially expressed genes was set to log_2_(treatment/CK fold-change) >  1 or  <  −1 (FDR < 0.01) (Wang et al., 2019). Ten genes were randomly selected to verify the reliability of transcriptome data by RT-PCR (Primer sequences in **Supplementary Table S1**).

### Identification of latent reservoirs-related genes and functional classification

The differentially expressed genes of *P. koraiensis* induced by FS*Bx* were designated PWN**-**induced genes. The genes that were upregulated in Latent Reservoirs and whose expression was unchanged or downregulated in the other three plant types were considered Latent Reservoirs-related genes.

Kyoto Encylopedia of Genes and Genomes (KEGG) enrichment analysis was performed on Latent Reservoirs-related genes (https://www.kegg.jp/). Through the analysis of the corresponding database, the KEGG information of Latent Reservoirs-related genes was obtained.

### Determination of H_2_O_2_ and EC content

The H_2_O_2_ content was detected by 3,3’-diaminobenzidine (DAB) histochemical staining. The stem segments of *P. koraiensis* branches were first sliced. For determination of the H_2_O_2_ content, the transverse sections were vacuumized in 50 mmol·L^−1^ Tris-HCl with 1 mg·ml^−1^ DAB solution; cultured in the dark at 25°C for 24 h; washed in 80% (V/V) ethanol at 70°C for 10 min; and fixed in lactic acid, phenol and water (V/V/V=1/1/1) (Zhang and Lin, 2019). The DAB-stained transverse sections were imaged by a microscope equipped with a digital camera.

According to previously described methods (Brennan and Frenkel, 1977; Liang et al., 2015), the content of H_2_O_2_ was determined by monitoring the absorbance at 415 nm (A_415_) of the titanium peroxide complex, with slight modifications. *P. koraiensis* branches (0.5 g) were immediately frozen in liquid nitrogen and then ground to a powder. The powder and 5 mL of cooled acetone were then mixed together in an ice bath. The mixture was centrifuged at 10,000 × g for 15 min (4°C) to obtain 1 mL of supernatant. To precipitate the titanium–hydrogen peroxide complex, 0.2 mL of titanium reagent (20% W/V) and 0.4 mL of ammonium solution were added. The reaction mixture was centrifuged at 10,000 × g for 10 min. The precipitate was dissolved in 5 mL of 2 M H_2_SO_4_, and the A_415_ was measured by an ultraviolet spectrophotometer. A standard response curve was generated according to the known H_2_O_2_ contents. The H_2_O_2_ content was estimated using a standard curve prepared with the H_2_O_2_ concentrations of standard solution. There were 3 biological replicates in the treatment and CK groups of 4 different types of *P. koraiensis*. The H_2_O_2_ content changes of *P. koraiensis* before and after PWNs infection were calculated.

Nontargeted metabolic analysis (BGI, Shenzhen, China) was used to detect the metabolites in the *P. koraiensis* branches and to determine the concentrations of metabolites in branches of different *P. koraiensis* individuals (Dunn et al., 2018). There were 3 biological replicates in the treatment and CK groups of 4 different types of *P. koraiensis*. The EC content was calculated by analyzing the nontarget metabolic data (Di Guida et al., 2016; Wen et al., 2017). The ratio of EC content is the ratio of EC content of *P. koraiensis* after PWN inoculation to that of CK group.

### RT-PCR validation

RT-PCR was used to measure the expression of 3 *ans* and 2 *anr* genes in 10 *P. koraiensis* individuals to further verify the transcriptome data (Primer sequences in **Supplementary Table S1**). According to the instructions of the manufacturer, a GoTaq Two-Step RT-PCR System Kit (Promega, Madison, Wisconsin, USA; directory number, A6010) and a Stratagene Mx3000P Qpcr system (Agilent Technologies, Santa Clara, California, USA) were used for RT-PCR (Chen et al., 2021a). Primer Premier 5 was used for designing primers, which are shown in **Supplementary Table S1**. The RT-PCR results were normalized (log_2_[fold-change]) with the *18S ribosomal RNA* and *Actin* as reference genes. Each reaction involved three independent repeated tests. The relative quantification method was used to calculate the data (Livak and Schmittgen, 2001).

### Gene network analysis

The correlation coefficient between genes was calculated by using the dynamic correlation heat map of the Omicshare (https://www.omicshare.com/tools/home/report/reporticawg.html). Select genes with correlation coefficients greater than 0.5. Cytoscape software was used to construct the network relationship between genes (Chen et al., 2021b).

### Statistical analysis

All data were analyzed by statistical software Graphpad Prism 8.0. Before the analysis, the normality and homogeneity of the variance were verified by Shapiro-Wilk and Levene tests, respectively. Data analysis was performed using one-way analysis of variance (ANOVA) based on the total number of factors in each experiment. Tukey’s post-test was used to compare differences between groups.

The stability of the two reference genes was analyzed using the analysis of the average level and variability of cycle thresholds (Cts) (Wei et al., 2020) and BestKeeper (Pfaffl et al., 2004). BestKeeper evaluates the stability of the reference genes based on the standard deviation (SD) and coefficient of variation (CV) of the Cts. RG with SD less than 1 is considered to be stably expressed. CV decreases with SD, indicating that reference gene is more stable (Pfaffl et al., 2004; Wei et al., 2020).

## Results

### PWD tolerance analysis of *P. koraiensis* individuals from Northeast China

*P. koraiensis* asymptomatic PWN carriers were found in Fushun City, Liaoning Province. These trees were infected during the feeding of the newly discovered vector sawyer beetle *Monochamus saltuarius* in the summer of the first year. The asymptomatic PWN carriers showed no symptoms until the following spring or summer (**Figure 1C**). This phenomenon is commonly referred to as “over-year death”.

**Figure 2:**
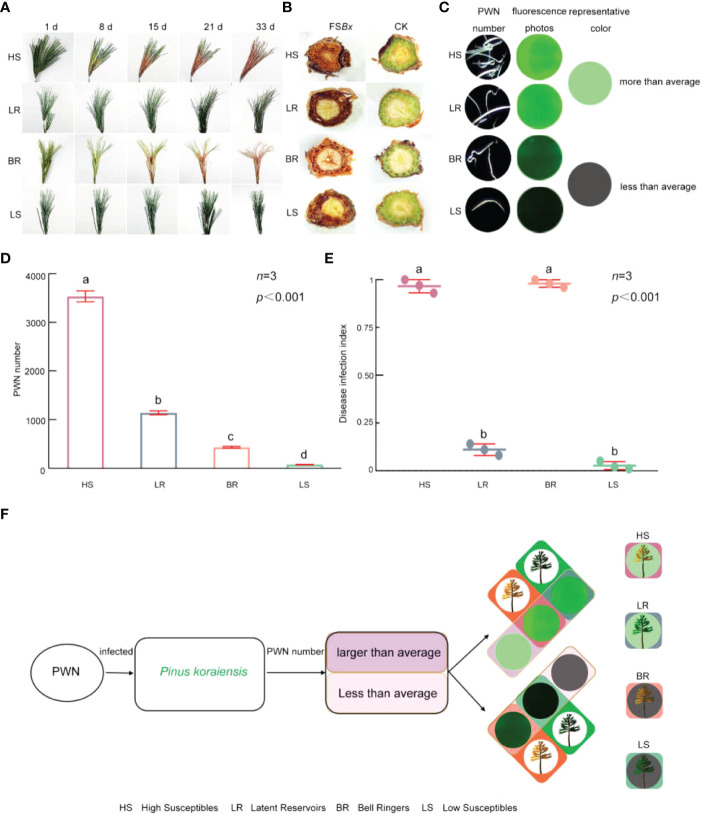
Characteristics of 4 types of *P. koraiensis* infected by PWN. **(A)** Symptoms of 4 types of *P. koraiensis* inoculated with PWN. **(B)** Transverse sections of PWN-infected branches at 33 dpi. **(C)** PWNs as seen *via* fluorescence microscopy images at 33 dpi. (When the number of PWNs exceeded 1,000, the green fluorescence was very obvious; thus, green was selected as the representative color of PWN-rich individuals, and gray represents individuals with fewer PWNs.) **(D)** Number of PWNs isolated from the 4 types of *P. koraiensis*. **(E)** Disease infection index of the 4 types of *P. koraiensis*. **(F)** Classification of the 4 types of *P. koraiensis*. Data in **(D, E)** were analyzed by one-way ANOVA followed by Tukey’s posthoc test, with different letters indicating statistically significant differences at 95% confidence. The data in the figures are means ± SE (*n*=3). Letter a: the maximum average number marked with the letter a Letter b: The maximum average is compared with the following averages. Where the difference is not significant, the letter a is marked until a significant difference is marked with the letter b Letter labeling followed by analogy. Where there is an identically marked letter, the difference is not significant; where there are different marked letters, the difference is significant.

PWNs were collected from diseased *P. koraiensis* in Fushun City, Liaoning Province, China (**Figure 1A**). *P. koraiensis* individuals were collected from 13 seed orchards in the Xiaoxing’an Mountains, Changbai Mountains and Qianshan Mountains in China (**Figure 1B**). The PWNs were cultured on *B. cinerea.* To identify the tolerance of different *P. koraiensis* individuals to PWN, two-year-old branches of 60 individuals were inoculated with PWN in a greenhouse in Liaoning Province, China. Sixty *P. koraiensis* individuals were classified on a scale from 0 to IV according to the disease infection index: 0, absence of symptoms; I, a quarter of the needles are chlorotic and brown; II, half of the needles are chlorotic and brown; III, only a quarter of the needles are healthy; and IV, all the needles are chlorotic and brown, and whole branches were withered. All 60 individuals were classified: 51 individuals were symptomatic (4 individuals classified as I; 8, as II; 9, as III; and 30, as IV), and 9 individuals were asymptomatic (classified as 0) (**Supplementary Table S2**). Among the 51 symptomatic individuals, the initial symptoms could be seen in 39 individuals at 7 dpi, in 8 individuals at 15 dpi and in 4 individuals at 21 dpi (**Supplementary Figure S1** and **Figure 2A**). Until 33 dpi, the most severely affected needles were chlorotic, brown and withered. The pathogenesis time of these 53 individuals was similar to that of other susceptible pine tree species (*P. thunbergii*, 19 days; *P. massoniana*, 29 days) (Chen et al., 2021b). However, asymptomatic individuals, known as asymptomatic PWN carriers, showed no symptoms at 33 dpi (**Supplementary Figure S1** and **Figure 2A**), while susceptible pines showed symptoms and died at 33 dpi. The needles of *P. koraiensis* in the CK group remained green and healthy for 33 days (**Supplementary Figure S1**).

Although transverse sections of all PWN-infected branches were brown due to oxidation, the branches of *P. koraiensis* showing symptoms were dark brown and appeared dried and shrunken at 33 dpi (**Figure 2B**). However, the sections of the branches of the trees in the CK group were green and healthy (**Figure 2B**).

PWNs in *P. koraiensis* branches were isolated and counted at 33 dpi (**Figures 2C, D** and **Supplementary Figure S2**). The number of PWNs isolated from *P. koraiensis* branches with different disease infection index were also different. On the basis of the number of PWNs (either greater or less than the average), the 60 individuals were divided into individuals with relatively more PWNs and individuals with fewer PWNs, which were 20 and 40, respectively (**Supplementary Table S3**). Among them, the disease infection index of two individuals with relatively more PWNs was 0.96 and 0.12, respectively, and the disease infection index of the two individuals with fewer PWNs was 0.98 and 0.02, respectively (**Figure 2E**).

The 60 *P. koraiensis* individuals, which were divided into 2 types according to the number of isolated PWNs, were further divided into 4 types due to the symptomatic or asymptomatic branches of *P. koraiensis* (**Figure 2E**). Individuals with more PWNs were divided into High Susceptibles (type 1) and Latent Reservoirs (type 2). Individuals with fewer PWNs included Bell Ringers (type 3) and Low Susceptibles (type 4). Among all the types, High Susceptibles type and Bell Ringers showed symptoms; however, Latent Reservoirs and Low Susceptibles type were asymptomatic (**Figure 2F**). Sixty *P. koraiensis* individuals including 17 High Susceptibles, 3 Latent Reservoirs, 34 Bell Ringers and 6 Low Susceptibles (**Supplementary Table S4**)

### Transcriptome analysis

*P. koraiensis* individuals from each of the 4 types of *P. koraiensis* were inoculated with PWN and ddH_2_O. *P. koraiensis* trees inoculated with PWN constituted the treatment group (Treatment 1, Treatment 2, Treatment 3, and Treatment 4), and *P. koraiensis* trees inoculated with ddH_2_O constituted the CK group (CK1, CK2, CK3, and CK4). To determine the characteristics of the 4 different types of *P. koraiensis*, 8 cDNA libraries (Treatment 1, CK1, Treatment 2, CK2, Treatment 3, CK3, Treatment 4, and CK4) of *P. koraiensis* were constructed and sequenced.

RNA sequencing of the 8 samples of *P. koraiensis* yielded an average of 6.39 Gb per sample (**Supplementary Table S5**). A total of 106,603 unigenes were identified by assembling all the samples and filtering for abundance. The total length, average length, N50 and GC contents were 121,574,412 bp, 1,140 bp, 1,861 bp and 43.62%, respectively. The dataset was deposited in the Sequence Read Archive (SRA, SRR18792142, SRR18792143, SRR18792144, SRR18792145, SRR18792146, SRR18792147, SRR18792148, and SRR18792149).

To verify the differentially expressed genes screened in the transcriptome data, 10 differentially expressed genes were randomly selected for RT-PCR. The results showed that the transcriptome data of 10 genes were consistent with the expression trend of RT-PCR data (**Supplementary Figure S3**). The above results show that the transcriptome data is reliable.

### Identification of differentially expressed genes and upregulated latent reservoirs-related genes

**Figure 3:**
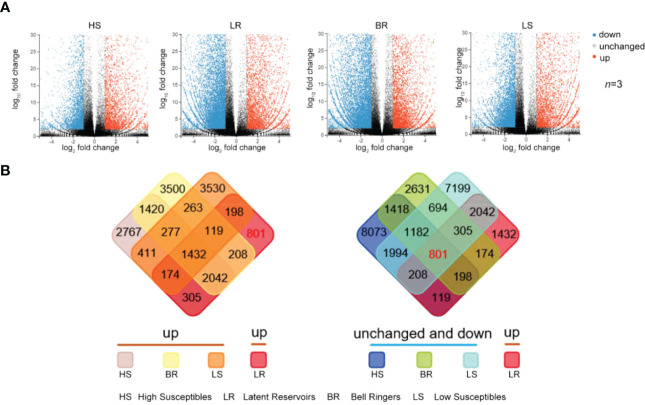
Identification of upregulated Latent Reservoirs-Related genes. **(A)** Scatter plots of genes expressed in response to PWN according to the library data of 4 types of trees (*t* test, n = 3). The expression level was normalized by the average number of FTPM according to the log_2_(fold change) of the normalized reads and the -log_10_(fold change) of the *P* value. The blue, red, and gray data points represent downregulated genes, upregulated genes or genes whose expression was unchanged. **(B)** Intersections of upregulated genes in Latent Reservoirs and all genes in the other three types.

The differentially expressed genes of the 4 types of *P. koraiensis* at 12 hour post-infection (hpi) were analyzed. Among them, 21,632 differentially expressed genes were found in High Susceptibles—8,828 upregulated genes and 12,804 downregulated genes. There were 9,261 upregulated genes and 6,042 downregulated genes in Bell Ringers. Moreover, there were 17,800 differentially expressed genes in Low Susceptibles (namely, 6,404 upregulated genes and 11,396 downregulated genes) and 8,472 differentially expressed genes in Latent Reservoirs (namely, 5,279 upregulated genes and 3,193 downregulated genes) (**Figure 3A** and **Supplementary Table S6**).

The intersection of differentially expressed genes among the 4 types of *P. koraiensis* was used to screen upregulated Latent Reservoirs-related genes, and 801 upregulated Latent Reservoirs-related genes were identified (**Figure 3B**, **Supplementary Figure S4**). Eight hundred and one genes were upregulated in Latent Reservoirs but downregulated or exhibited no change in expression in the other three types (**Figure 3B**, **Supplementary Figure S4**). According to criterion of fragments per kilobase of transcript per million mapped reads (FPKM) > 1, 528 of the 801 genes were included.

### Functional and pathway enrichment analyses of candidate genes

**Figure 4:**
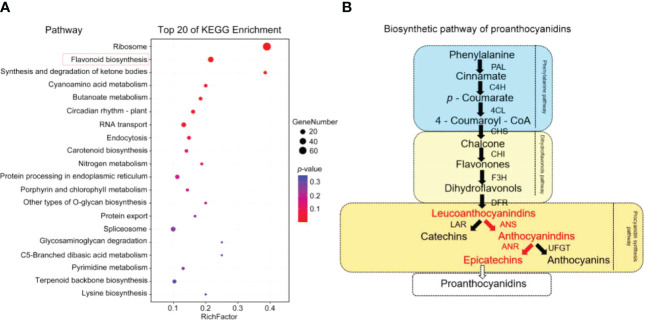
KEGG enrichment analysis of candidate genes and the main enriched pathways. **(A)** KEGG enrichment analysis map. **(B)** Biosynthesis pathway of procyanidins (the red text indicates the components of the synthesis pathway of EC).

After PWNs infected *P. koraiensis*, the cross-sectional area of *P. koraiensis* transverse sections was reddish brown. This sign is consistent with the symptoms of plant oxidation, considering that oxidation occurs in *P. koraiensis* after PWNs infection. So, our focus is on oxidation. Among the 528 upregulated Latent Reservoirs-related genes, 268 were enriched in 83 KEGG pathways (**Figure 4A** and **Supplementary Table S7**), mainly those involving ribosomes (ko03010) and flavonoid biosynthesis (ko00941) (**Figure 4A**). Flavonoids play an important role in plant growth and development and in improving plant stress tolerance. Therefore, 25 genes enriched in the flavonoid pathway (**Supplementary Figure S5**) according to the KEGG enrichment analysis were selected for further study.

Further KEGG analysis of the 25 genes showed that there were 10 chalcone-related genes, 5 anthocyanin-related genes, 4 naringenin 3-dioxygenase genes, 2 bifunctional dihydroflavonol 4-reductase genes, 2 flavonoid 3’,5’-hydroxylase genes, 1 caffeoyl-CoA O-methyltransferase gene and 1 gibberellin-44 dioxygenase gene. There were 15 genes involved in the procyanidin synthesis pathway. The procyanidin synthesis pathway is an important branch of the flavonoid synthesis pathway (**Supplementary Figure S5**). The process of procyanidin synthesis can be divided into three main stages (**Figure 4B**). Eleven enzymes are involved in this synthesis process, including ANS and ANR (**Figure 4B**).

**Figure 5:**
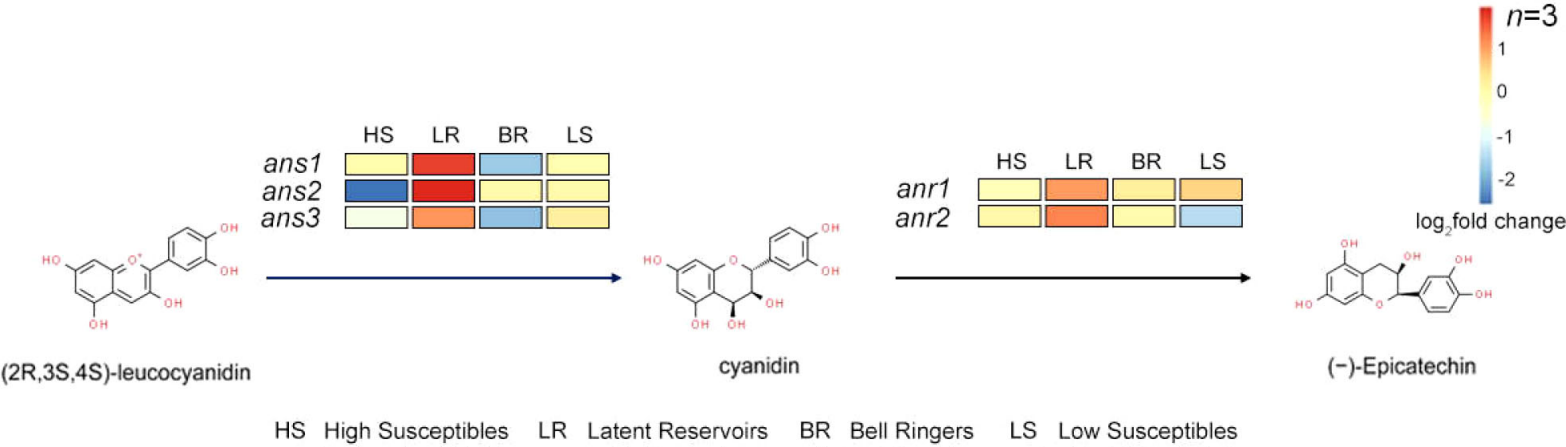
Changes in the expression of EC synthesis-related pathway genes in 4 *P. koraiensis* plant types. Pathway of EC synthesis. (The color-coded blocks represent the expression levels of 3 *ans* and 2 *anr* genes in High Susceptibles, Latent Reservoirs, Bell Ringers, and Low Susceptibles. The pathway was drawn according to information in literature and the KEGG database results.).

Five anthocyanin-related genes were identified: 3 anthocyanin synthase (*ans*) genes and 2 anthocyanin reductase (*anr*) genes. In the procyanidin synthesis pathway, ANS converts leucoanthocyanidins into cyanidins, after which cyanidins are converted into EC through ANR (**Figures 4B**, **5**). By analyzing the expression of these 5 genes of the 4 types of *P. koraiensis* at 12 hpi, we found that the expression of these 5 genes was upregulated in Latent Reservoirs (**Figure 5**). However, the expression of *ans1*, *ans2*, *ans3* and *anr2* was downregulated or unchanged in the others. *Anr1* expression was unchanged in the other three *P. koraiensis* types.

The expression of 5 genes of 4 types of *P. koraiensis* was further analyzed at 24 hpi, 48 hpi and 72 hpi. It was found that at 24 hpi, 48 hpi and 72 hpi, the expression levels of 5 genes (except for *ans3* and *anr2* at 24 hpi, *ans1* at 48 hpi and *ans2* at 72 hpi)were up-regulated in Latent Reservoirs. It was also found that the expression of these 5 genes (except *ans1* and *anr2* in High Susceptibles) was significantly down-regulated in the other three types of *P. koraiensis* at 24 hpi and 48 hpi. At 72 hpi, *ans1* in High Susceptibles, *ans1* and *anr2* in Bell Ringers and *ans1*, *ans3* and *anr2* in Low Susceptibles were up-regulated, and other genes were down-regulated in the three types (**Supplementary Figure S6**). This result was consistent with the results obtained at 12 hpi, again indicating that the expression of 5 EC synthesis-related genes was always up-regulated in Latent Reservoirs.

### H_2_O_2_ contents and EC contents of 4 types of *P. koraiensis*

**Figure 6:**
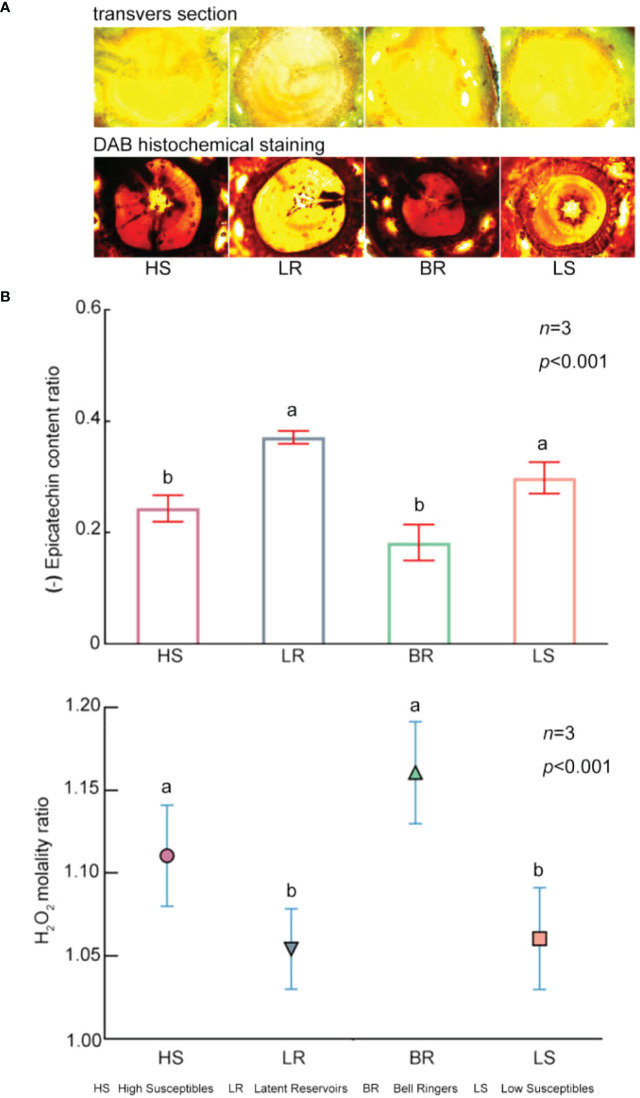
Contents of EC and H_2_O_2_ in 4 types of *P. koraiensis*. **(A)** Changes in H_2_O_2_ levels in 4 types of *P. koraiensis*, as revealed by DAB histochemical staining at 12 hpi. **(B)** H_2_O_2_ molality ratio and EC content ratio in the 4 types of *P. koraiensis* at 12 hpi. (The histogram represents the EC content ratio, and the scatter plots with different shapes represent the H_2_O_2_ molality ratio). Data in **(B)** were analyzed by one-way ANOVA followed by Tukey’s posthoc test, with different letters indicating statistically significant differences at 95% confidence. The data in the figures are means ± SE (*n*=3). Letter a: the maximum average number marked with the letter a Letter b: The maximum average is compared with the following averages. Where the difference is not significant, the letter a is marked until a significant difference is marked with the letter b Letter labeling followed by analogy. Where there is an identically marked letter, the difference is not significant; where there are different marked letters, the difference is significant.

According to DAB histochemical staining, the stained transverse sections of High Susceptibles and Bell Ringers were dark brown, while those of Low Susceptibles and Latent Reservoirs were light brown (**Figure 6A**). These results indicated that after inoculation with PWN, the content of H_2_O_2_ in High Susceptibles and Bell Ringers was higher than that in Low Susceptibles and Latent Reservoirs.

Additionally, the content of H_2_O_2_ in *P. koraiensis* was measured using a spectrophotometer. Among the 4 types of *P. koraiensis*, the content of H_2_O_2_ at 12 hpi was 1.11 times, 1.05 times, 1.16 times and 1.06 times higher than that of the trees in the CK group (**Figure 6B**, **Supplementary Table S8**). The results of the content assays were consistent with the DAB staining results.

The content of EC was significantly lower in High Susceptibles, Bell Ringers, Low Susceptibles and Latent Reservoirs at 12 hpi than in the CK plants by approximately 0.24 times, 0.18 times, 0.29 times, and 0.37 times, respectively (**Figure 6B**, **Supplementary Table S9**). This showed that EC was consumed when *P. koraiensis* was infected by PWN.

The content of H_2_O_2_ was lower and the content of EC was higher in the asymptomatic PWN carriers *P. koraiensis* than in the symptomatic *P. koraiensis*. Among the different types, the content of EC was the highest in Latent Reservoirs, and the content of H_2_O_2_ was the lowest. These results showed that there was increased antioxidant activity in Latent Reservoirs.

### Anthocyanin-related gene expression levels in other *P. koraiensis* individuals

Correlation analysis of anthocyanin-related genes FPKM and EC content showed that except for *ans2* (the correlation coefficient is -0.35) and *ans3* (the correlation coefficient is -0.02), the other 3 genes were positively correlated with EC content, with correlation coefficients of 0.19, 0.20, and 0.35, respectively (**Supplementary Figure S7**).To further verify the expression levels of 5 anthocyanin-related genes in different *P. koraiensis* individuals, 10 individuals were selected from the original 60 P*. koraiensis* individuals and named Nos. 1 to 10, respectively. There were 2 individuals of High Susceptibles (No. 1 and No. 2), 2 individual Latent Reservoirs (No. 3 and No. 4), 4 individuals of Bell Ringers (No. 5, No. 6, No. 7 and No. 8) and 2 individuals of Low Susceptibles (No. 9 and No. 10).

Ct is the number of cycles that the reaction curve intersects with the threshold line. It represents the number of cycles required to detect a true signal from a sample. To analyze the expression stability of the two reference genes, the Cts of 20 samples were analyzed with analysis of the average level and variability of Cts and BestKeeper. Average Cts of 18.74 (*Actin*) and 17.14 (*18S ribosomal RNA*). The change of Cts of *Actin* is larger than that of *18S ribosomal RNA* (**Supplementary Figure S8A**). BestKeeper calculated SD values of *Actin* and *18S ribosomal RNA* were less than 1, indicating that both reference genes are stable. CV decreases with SD, indicating that reference gene is more stable. The CV value of *18S ribosomal RNA* (3.69%) was less than that of *Actin* (4.82%) (**Supplementary Table S10**), indicating that *18S ribosomal RNA* was more stable as a reference gene. When *18S ribosomal RNA* and *Actin* were used as reference genes respectively, we observed that 3 *ans* genes and 2 *anr* genes had similar expression trends (**Supplementary Figure S8B**). Therefore, *18S ribosomal RNA* was selected as the reference gene.

**Figure 7:**
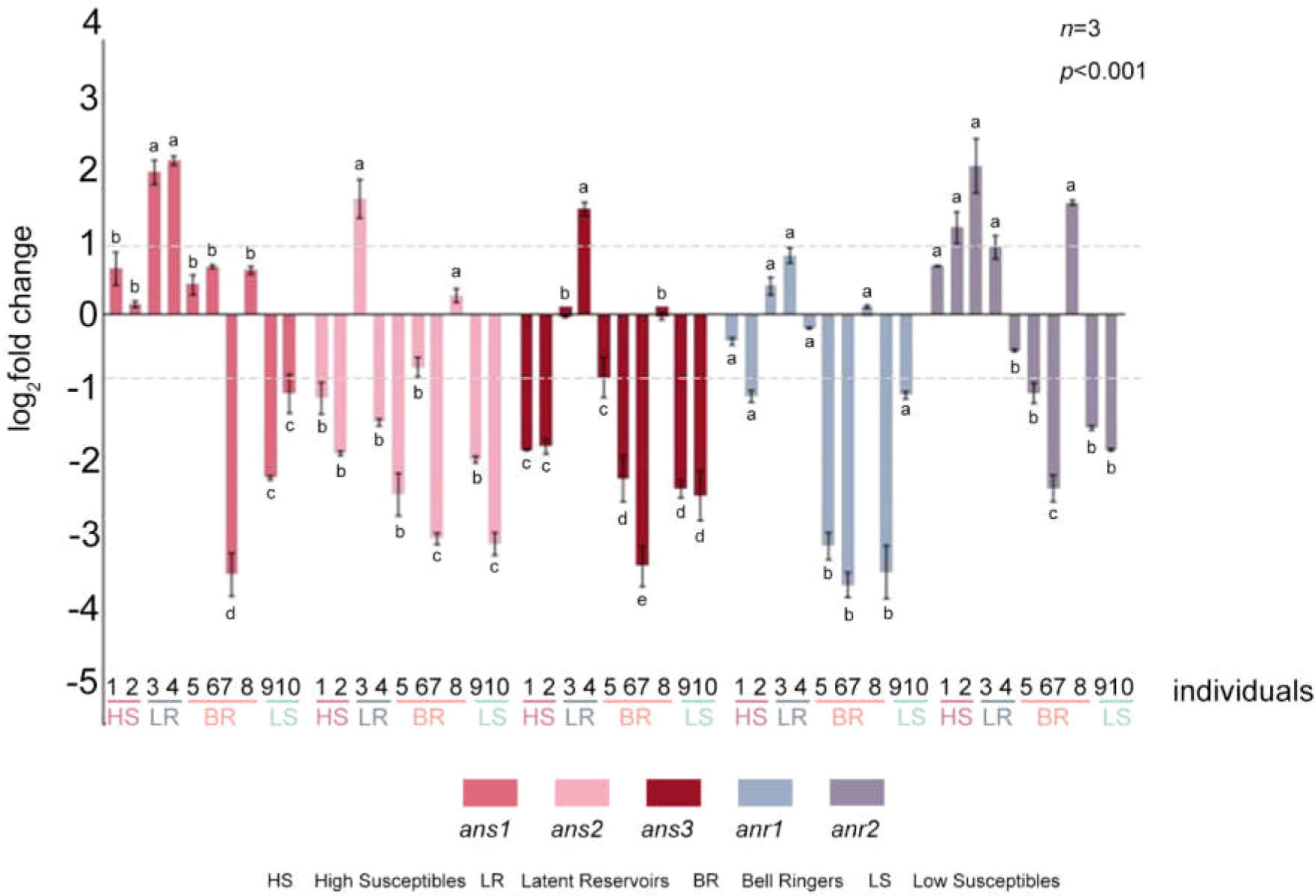
Expression levels of 3 *ans* and 2 *anr* genes in 10 *P. koraiensis* individuals at 12 hpi. Data were analyzed by one-way ANOVA followed by Tukey’s posthoc test, with different letters indicating statistically significant differences at 95% confidence. The data in the figure are means ± SE (*n*=3). Letter a: the maximum average number marked with the letter a Letter b: The maximum average is compared with the following averages. Where the difference is not significant, the letter a is marked until a significant difference is marked with the letter b Letter labeling followed by analogy. Where there is an identically marked letter, the difference is not significant; where there are different marked letters, the difference is significant.

The RT-PCR results indicated that *ans1*, *ans2* and *anr2* were significantly higher in No. 3 and that *ans1*, *ans3* and *anr2* were significantly higher in No. 4 at 12 hpi compared with those in the CK group (**Figure 7**, **Supplementary Figure S8**). Moreover, compared with that in the CK group, the expression of 3 *ans* and 2 *anr* genes was unchanged or was significantly lower at 12 hpi in Nos. 1, 5, 6, 7, 9, and 10. However, *anr2* was significantly higher in No. 2 and No. 8 at 12 hpi than in it was in the CK group (**Figure 7**, **Supplementary Figure S8**). These findings showed that both *ans* and *anr* were upregulated in Latent Reservoirs. Plants in which *ans* or *anr* were only upregulated were not Latent Reservoirs.

The correlation between the expression of 5 genes in increased 10 individuals of 4 types and the expression of 5 genes in 4 types of transcriptome sequencing was analyzed. The results showed that the expression of 5 genes in the same type of *P. koraiensis* was highly correlated, except for No. 8 (Correlation coefficient, 0.28), the correlation coefficients were greater than 0.45 (**Supplementary Figure S9**).

## Discussion

*P. koraiensis* is an evergreen coniferous species, which is an important native and precious forest species in Northeast China, far East of Russia and Korean Peninsula (Li et al., 2020). *P. koraiensis* has great ecological and economic value and plays an important role in both ecological environment construction and economic development in Northeast China (Li et al., 2022a). For centuries, owing to its excellent wood properties and nutritional value, *P. koraiensis* has been widely used as a major source of high-quality timber, natural medicine and edible pine nuts (Kaviriri et al., 2020). Additionally, *P. koraiensis* is also the dominant tree species of mixed forests in the cold temperate zone of China (mainly distributed in the Changbai Mountains and Xiaoxing’an Mountains in Northeast China). This mixed forest in Northeast China is critical to China because it is an important base for wood supply, an important reservoir of biodiversity, a potential carbon sequestration area, a destination for ecotourism and a broad ecological barrier in Northeast China (Yu et al., 2011; Kaviriri et al., 2020). In 2013, *P. koraiensis* was listed as a vulnerable species by the International Union for Conservation of Nature (Kaviriri et al., 2020). According to an announcement of the Ministry of Agriculture and Rural Affairs of the China State Forestry and Grassland Administration (No. 15 of 2021), *P. koraiensis* is listed as a key protected wild plant in secondary countries. Therefore, to protect and utilize precious *P. koraiensis* wild resources, it is necessary to protect *P. koraiensis* from PWN. For this, the most important thing is to understand the response mechanism of *P. koraiensis* asymptomatic PWN carriers to PWN infection.

PWN undergoes an obvious incubation period in North America, the process of which is influenced by the host and environment (Dropkin, 1981; Wingfield, 1982; Halik and Bergdahl, 1994; Li et al., 2021). During the field investigation of *P. koraiensis* in Liaoning Province, over-year death of *P. koraiensis* after PWN infection was found. In the experiment with *P. koraiensis* individuals in response to PWN infection, a few branches of *P. koraiensis* individuals remained asymptomatic at 33 dpi after PWN infection. According to the symptoms of *P. koraiensis* and the number of PWNs, *P. koraiensis* individuals were divided into 4 different types. Among them, Low Susceptibles and Latent Reservoirs were asymptomatic PWN carriers. Low Susceptibles had a few PWNs that had little impact. Latent Reservoirs had numerous PWNs that could become sources of infection the next year, which is very dangerous. There was no obvious difference between the *P. koraiensis* asymptomatic PWN carriers and healthy *P. koraiensis* trees. *P. koraiensis* asymptomatic PWN carriers are easily overlooked when *P. koraiensis* trees in a stand damaged by PWN are cut down. In the prevention and control of PWD, diseased wood cleaning is one of the main measures. It becomes particularly important to identify Latent Reservoirs in forest stands (Futai, 2013; Li et al., 2021). Therefore, it is critical to study the asymptomatic mechanisms of *P. koraiensis* Latent Reservoirs in the process of controlling PWD.

At present, in the study of plant nematodes, the disease tolerance of host plants is defined as the ability to withstand or recover from the damage caused by nematodes (Trudgill, 1991; Jones et al., 2008). Pine trees resistant to PWN have been shown to display a HR at the PWN infection site (Jones et al., 2008). In the experiment with *P. koraiensis* individuals in response to PWN infection, the content of H_2_O_2_ was higher in the 4 types of *P. koraiensis* at 12 hpi than in the trees in the CK group. These results showed that when PWN infected *P. koraiensis*, the *P. koraiensis* trees produced oxidation reactions to protect themselves from PWN. However, when ROS accumulate to a certain extent, they will cause a HR (Gill and Tuteja, 2010; Ren et al., 2021). However, the HR of pine trees cannot inhibit the growth or reproduction of PWN because of the mycetophagous phases of these organisms (Tsai et al., 2016). There were many PWNs in Latent Reservoirs, but the content of H_2_O_2_ was lower than it was in the other three types. These results indicate that antioxidative reactions occurred in Latent Reservoirs.

Further study of the Latent Reservoirs showed that genes involved the metabolite pathway were differentially upregulated compared with those of the other three plant types, and there were more flavonoid pathway genes that were upregulated. Among the upregulated genes involved in the flavonoid pathway, 3 *ans* and 2 *anr* genes were related to EC synthesis. The stable expression of *anr* in various plants is related to the accumulation of procyanidins and EC (Pang et al., 2007; Szankowski et al., 2009; Pang et al., 2013; Hammerbacher et al., 2014). ANS is an enzyme that plays a key role downstream of the anthocyanin biosynthesis pathway (Liu et al., 2021). *Ans* and *anr* are also related to plant antioxidative activity (Wang et al., 2013; Liu et al., 2021). As flavonoid, EC, like other flavonoid, contributes to the color change and sexual reproduction of flowers and fruits. EC is also a stress resistance substance and antioxidant, which is important to improving plant resistance (Ullah et al., 2017).

The EC content in all *P. koraiensis* trees infected with PWN was lower than that in the CK group. However, the content of EC in Latent Reservoirs was higher than that in the other three types. In Latent Reservoirs, the expression of the *ans* and *anr* genes was upregulated at 12 hpi, indicating that when PWNs infected Latent Reservoirs, the EC synthesis pathway was always synthesizing EC. The transcript levels of *anr* were significantly correlated with EC content in *Vitis vinifera* and *Malus domestica* (Gagné et al., 2009; Han et al., 2012; Liao et al., 2015), and this was verified in the present study. As important dietary antioxidant, EC is a potent ROS scavenger involved in the redox modulation of cells (Fraga et al., 2018; Zhang et al., 2018). The low content of EC indicated that they participate in the antioxidant process of all *P. koraiensis* trees. However, in symptomatic *P. koraiensis* trees, EC did not have an antioxidant effect. Latent Reservoirs did not show typical symptoms of ROS, which showed that EC exhibited antioxidant activity. We speculated that EC could reduce the content of H_2_O_2_ to improve the antioxidant activity of *P. koraiensis*.

In apples, the antioxidant activity inhibits the accumulation of mycotoxin accumulation. The apple cultivars with the strongest antioxidant activity have the higher content of catechin and EC (Lončarić et al., 2021). It has also been shown that the polyphenols from *Rheum* roots, which include EC, can inhibit the growth of fungal and oomycete plant pathogens and induce disease resistance in plants (Gillmeister et al., 2019). The above studies show that EC contributes to the antioxidant activity of plants and also plays an important role in the antifungal process of plants (Xin et al., 2020). Antioxidant processes involving EC were also found to exist in *P. koraiensis* after PWN infestation in this study.

The genes and KEGG pathways involved in EC were analyzed, and the related network diagram was found. Three *ans* genes and 2 *anr* genes were screened for genes highly related to them. Genes with weights greater than 0.2 are selected and their network data is output to Cytoscape. The whole network contains 30 regulatory relationships of 32 genes (**Supplementary Figure S10**). Thirty-two genes were enriched in 12 different KEGG pathways. The 12 pathways include MAPK pathway (ko04010, ko04011, ko04013, and ko04016), NF-kappa B signaling pathway (ko04064), flavonoid biosynthesis (ko00941), cytochrome P450 (ko00980 and ko00982), Calcium signaling pathway (ko04020), plant hormone signal transduction (ko04075), NOD-like receptor signaling pathway (ko04621), and plant-pathogen interaction (ko04626). MAPK, NF-kappa B, NOD-like receptor signaling pathway and cytochrome P450 are related to cellular redox (Fraga et al., 2018). Therefore, it is speculated that EC in Latent Reservoirs achieves antioxidant effects by affecting these pathways.

In this study, according to the changes of H_2_O_2_ content of four types *P. koraiensis* after PWNs infection, it was found that the changes of H_2_O_2_ content in *P. koraiensis* were different after PWNs infection. Among them, the H_2_O_2_ content of Latent Reservoirs and Low Susceptibles changed little after PWNs infection. Simultaneously, we found that EC content decreased in the four types after PWNs infection, and Latent Reservoirs and Low Susceptibles decreased less. EC has direct antioxidant activity (Fraga et al., 2018). Therefore, we believe that EC is involved in the antioxidant process of *P. koraiensis* after PWNs infection. In Latent Reservoirs, the antioxidant process decreased symptoms of PWD. Studies have shown that regulating the antioxidant system of *P. pinaster* can improve the tolerance of *P. pinaster* to PWN (Nunes da Silva et al., 2021). Therefore, the importance of the antioxidant pathway in the host plant response to PWN infection is worthy of further study.

Our results showed that the contents of H_2_O_2_ and EC in *P. koraiensis* were changed after PWNs infection. It indicated that oxidation and antioxidation reaction occurred in *P. koraiensis* after PWNs infection. Among them, only the antioxidant process involving EC in Latent Reservoirs decreased symptoms of PWD. Various inanimate and biological agents disrupt the balance between cellular oxidants and antioxidants (Šoln, K et al., 2022). Identifying Latent Reservoirs through oxidation and antioxidant processes becomes particularly important. It is more meaningful if Latent Reservoirs can be identified in the nursery through oxidation and antioxidant parameters. In the future, our research group will classify *P. koraiensis* by inoculating PWNs on a large number of individual branches in the nursery, in order to obtain a large number of four types of *P. koraiensis* genome information. Identifying four types through the differences in SNPs and SSRs in different types will be our future research object. Rapid identification of Latent Reservoirs will facilitate the control of PWN in Northeast China.

In conclusion, there are different degrees of disease tolerance among *P. koraiensis* individuals infected with PWN. Additionally, the molecular mechanisms of oxidation and antioxidation of symptomatic and asymptomatic PWN carriers of *P. koraiensis* were also studied. These results could help to increase the understanding of Latent Reservoirs and provide a theoretical basis for the control of PWD.

## Data availability statement

The data presented in the study are deposited in the NCBI repository (https://www.ncbi.nlm.nih.gov/), accession numbers SRR18792142, SRR18792143, SRR18792144, SRR18792145, SRR18792146, SRR18792147, SRR18792148, and SRR18792149.

## Author contributions

Conceived and designed the experiments: FW, and DLL. Performed the experiments: RZZ, JNW, RX, and FW. Analyzed the data: FW, and RZZ. Contributed reagents/materials/analytical tools: FW, and RZZ. Wrote the paper: RZZ, DLL and FW. All authors have read and approved the manuscript.

## References

[B1] Apel K. Hirt H. (2004). Reactive oxygen species: metabolism, oxidative stress, and signal transduction. Annu. Rev. Plant Biol. 55, 373–399. doi: 10.1146/annurev.arplant.55.031903.141701 15377225

[B2] Benjamini Y. Yekutieli Y. (2001). The control of the false discovery rate in multiple testing under dependency. Ann. Stat 29 (4), 1165–1188. doi: 10.1214/aos/1013699998

[B3] Brennan T. Frenkel C. (1977). Involvement of hydrogen peroxide in the regulation of senescence in pear. Plant Physiol. 59 (3), 411–416. doi: 10.1104/pp.59.3.411 16659863 PMC542414

[B4] Chen Z. Liang J. Zhang C. Rodrigues C. J. Jr. (2006). Epicatechin and catechin may prevent coffee berry disease by inhibition of appressorial melanization of *Colletotrichum kahawae* . Biotechnol. Lett. 28 (20), 1637–1640. doi: 10.1007/s10529-006-9135-2 16955359

[B5] Chen Q. Zhang R. Li D. Wang F. (2021a). Integrating transcriptome and coexpression network analyses to characterize salicylic acid-and jasmonic acid-related genes in tolerant poplars infected with rust. Int. J. Mol. Sci. 22 (9), 5001. doi: 10.3390/ijms22095001 34066822 PMC8125932

[B6] Chen Q. Zhang R. Li D. Wang F. (2021b). Transcriptomic and coexpression network analyses revealed pine *Chalcone synthase* genes sssociated with pine wood nematode infection. Int. J. Mol. Sci. 22 (20), 11195. doi: 10.3390/ijms222011195 34681852 PMC8540587

[B7] Di Guida R. Engel J. Allwood J. W. Weber R. J. M. Jones M. R. Sommer U. . (2016). Non-targeted UHPLC-MS metabolomic data processing methods: a comparative investigation of normalisation, missing value imputation, transformation and scaling. Metabolomics 12, 93. doi: 10.1007/s11306-016-1030-9 27123000 PMC4831991

[B8] Dropkin V. H. (1981). Pinewood nematode: a threat to U.S. forests? Plant Dis. (USA) 65 (12), 1022–1027. doi: 10.1094/PD-65-1022

[B9] Dunn W. B. David B. Paul B. Eva Z. Sue F. M. Nadine A. . (2018). Procedures for large-scale metabolic profiling of serum and plasma using gas chromatography and liquid chromatography coupled to mass spectrometry. Nat. Protoc. 6 (7), 1060–1083. doi: 10.1038/nprot.2011.335 21720319

[B10] Fraga C. G. Oteiza P. I. Galleano M. (2018). Plant bioactives and redox signaling: (-)-Epicatechin as a paradigm. Mol. Aspects Med. 61, 31–40. doi: 10.1016/j.mam.2018.01.007 29421170

[B11] Futai (2013). Pine wood nematode, *Bursaphelenchus xylophilus* . Annu. Rev. Phytopathol. 51 (-), 61–83. doi: 10.1146/annurev-phyto-081211-172910 23663004

[B12] Gagné S. Lacampagne S. Claisse O. Gény L. (2009). Leucoanthocyanidin reductase and anthocyanidin reductase gene expression and activity in flowers, young berries and skins of *Vitis vinifera* l. cv. Cabernet-sauvignon during development. Plant Physiol. Biochem. 47 (4), 282–290. doi: 10.1016/j.plaphy.2008.12.004 19136268

[B13] Gillmeister M. Ballert S. Raschke A. Geistlinger J. Kabrodt K. Baltruschat H. . (2019). Polyphenols from *Rheum* roots inhibit growth of fungal and oomycete phytopathogens and induce plant disease resistance. Plant Dis. 103 (7), 1674–1684. doi: 10.1094/PDIS-07-18-1168-RE 31095470

[B14] Gill S. S. Tuteja N. (2010). Reactive oxygen species and antioxidant machinery in abiotic stress tolerance in crop plants. Plant Physiol. Biochem. 48 (12), 909–930. doi: 10.1016/j.plaphy.2010.08.016 20870416

[B15] Goupil P. Peghaire E. Benouaret R. Richard C. Sleiman M. El Alaoui H. . (2020). Relationships between plant defense inducer activities and molecular structure of gallomolecules. J. Agric. Food Chem. 68 (52), 15409–15417. doi: 10.1021/acs.jafc.0c05719 33337882

[B16] Halik S. Bergdahl D. (1994). Long-term survival of *Bursaphelenchus xylophilus* in living *Pinus sylvestris* in an established plantation. Eur. J. For. Pathol. 24 (6-7), 357–363. doi: 10.1111/j.1439-0329.1994.tb00829.x

[B17] Hammerbacher A. Paetz C. Wright L. P. Fischer T. C. Bohlmann J. Davis A. J. . (2014). Flavan-3-ols in Norway spruce: biosynthesis, accumulation, and function in response to attack by the bark beetle-associated fungus *Ceratocystis polonica* . Plant Physiol. 164 (4), 2107–2122. doi: 10.1104/pp.113.232389 24550241 PMC3982766

[B18] Han Y. Vimolmangkang S. Soria-Guerra R. E. Korban S. S. (2012). Introduction of apple *ANR* genes into tobacco inhibits expression of both *CHI* and *DFR* genes in flowers, leading to loss of anthocyanin. J. Exp. Bot. 63 (7), 2437–2447. doi: 10.1093/jxb/err415 22238451 PMC3346214

[B19] Heath M. C. (2000). Hypersensitive response-related death. Plant Mol. Biol. 44 (3), 321–334. doi: 10.1023/a:1026592509060 11199391

[B20] Hou Z. Shi F. Ge S. Tao J. Zhang S. (2021). Comparative transcriptome analysis of the newly discovered insect vector of the pine wood nematode in China, revealing putative genes related to host plant adaptation. BMC Genomics 22 (1). doi: 10.1186/s12864-021-07498-1 PMC7968331 33726671

[B21] Jones J. D. G. Dangl J. L. (2006). The plant immune system. Nature 444, 323–329. doi: 10.1038/nature05286 17108957

[B22] Jones J. T. Moens M. Mota M. Li H. Kikuchi T. (2008). *Bursaphelenchus xylophilus*: opportunities in comparative genomics and molecular host-parasite interactions. Mol. Plant Pathol. 9 (3), 357–368. doi: 10.1111/j.1364-3703.2007.00461.x 18705876 PMC6640334

[B23] Kaviriri D. K. Zhang Q. Zhang X. Jiang L. Zhang J. Wang J. . (2020). Phenotypic variability and genetic diversity in a *Pinus koraiensis* clonal trial in northeastern China. Genes 11 (6), 672. doi: 10.3390/genes11060673 32575537 PMC7348814

[B24] Kenichi Y. Takuma T. Natsumi K. Masabumi K. Levia D. F. Daisuke K. . (2018). Pine wilt disease causes cavitation around the resin canals and irrecoverable xylem conduit dysfunction. J. Exp. Bot. 69 (3), 589–602. doi: 10.1093/jxb/erx417 29240955

[B25] Lamb C. Dixon R. A. (1997). The oxidative burst in plant disease resistance. Annu. Rev. Plant Physiol. Plant Mol. Biol. 48, 251–275. doi: 10.1146/annurev.arplant.48.1.251 15012264

[B26] Liang S. Li G. Zhang X. Ren A. Gao T. Zhao M. (2015). The regulation of methyl jasmonate on hyphal branching and GA biosynthesis in *Ganoderma lucidum* partly *via* ROS generated by NADPH oxidase. Fungal Genet. Biol. 81 (12), 201–211. doi: 10.1016/j.fgb.2014.12.002 25512263

[B27] Liao L. Vimolmangkang S. Wei G. Zhou H. Korban S. S. Han Y. (2015). Molecular characterization of genes encoding leucoanthocyanidin reductase involved in proanthocyanidin biosynthesis in apple. Front. Plant Sci. 6 (243). doi: 10.3389/fpls.2015.00243 PMC4392590 25914714

[B28] Li X. Cai K. Zhao Q. Li H. Wang X. Tigabu M. . (2022a). Morphological and comparative transcriptome analysis of three species of five-needle pines: insights into phenotypic evolution and phylogeny. Front. Plant Sci. 13. doi: 10.3389/fpls.2022.795631 PMC8866173 35222462

[B29] Li Y. Chen Y. Wang X. Liu Z. Zhu T. Zhang X. (2021). Latent infection of pine wilt disease. J. OF Beijing FORESTRY Univ. 43 (09), 14–18. doi: 10.12171/j.1000-1522.20210218

[B30] Li X. Liu X. Wei J. Li Y. Tigabu M. Zhao X. (2020). Development and transferability of EST-SSR markers for *Pinus koraiensis* from cold-stressed transcriptome through illumina sequencing. Genes 11 (5), 500. doi: 10.3390/genes11050500 32370137 PMC7291311

[B31] Li Y. Zhang X. (2018). Analysis on the trend of invasion and expansion of Bursaphelenchus xylophilus. Forest Pest and Disease 37 (5), 1–4. doi: 10.3969/j.issn.1671-0886.2018.05.001

[B32] Liu K. Wang M. Xin H. Zhang H. Cong R. Huang D. (2021). Anthocyanin biosynthesis and regulate mechanisms in plants: A review. Chin. Agric. Sci. Bull. 37 (14), 41–51. doi: 10.11924/j.issn.1000-6850.casb2020-0390

[B33] Livak K. J. Schmittgen T. D. (2001). Analysis of relative gene expression data using real-time quantitative PCR and the 2^-^ * ^ΔΔC^ * ^T^ method. Methods 25 (4), 402–408. doi: 10.1006/meth.2001.1262 11846609

[B34] Li D. Wang F. Wang C. Zou L. Wang Z. Chen Q. . (2016). MicroRNA-mediated susceptible poplar gene expression regulation associated with the infection of virulent *Melampsora larici-populina* . BMC Genomics 17 (1), 59. doi: 10.1186/s12864-015-2286-6 26768277 PMC4714501

[B35] Li X. Zhang J. Lin S. Xing Y. Zhang X. Ye M. . (2022b). (+)-catechin, epicatechin and epigallocatechin gallate are important inducible defensive compounds against ectropis grisescens in tea plants. Plant Cell Environ. 45 (2), 496–511. doi: 10.1111/pce.14216 34719788

[B36] Lončarić A. Šarkanj B. Gotal A. M. Kovač M. Nevistić A. Fruk G. . (2021). *Penicillium expansum* impact and patulin accumulation on conventional and traditional apple cultivars. Toxins (Basel) 13 (10), 703. doi: 10.3390/toxins13100703 34678996 PMC8541162

[B37] Mamiya Y. (1983). Pathology of the pine wilt disease caused by *Bursaphelenchus xylophilus* . Annu. Rev. Phytopathol. 21, 201–220. doi: 10.1146/annurev.py.21.090183.001221 25946434

[B38] Mittler R. Vanderauwera S. Gollery M. Breusegem F. V. (2004). Reactive oxygen gene network of plants. Trends Plant Sci. 9 (10), 490–498. doi: 10.1016/j.tplants.2004.08.009 15465684

[B39] Modesto I. Sterck L. Arbona V. Gómez-Cadenas A. Miguel C. M. (2021). Insights into the mechanisms implicated in *Pinus pinaster* resistance to pinewood nematode. Front. Plant Sci. 12. doi: 10.3389/fpls.2021.690857 PMC8222992 34178007

[B40] Nanda A. K. Andrio E. Marino D. (2010). Reactive oxygen species during plant-microorganism early interactions. J. Integr. Plant Biol. 52 (2), 195–204. doi: 10.1111/j.1744-7909.2010.00933.x 20377681

[B41] Nunes da Silva M. Santos C. S. Cruz A. López-Villamor A. Vasconcelos M. W. (2021). Chitosan increases *Pinus pinaster* tolerance to the pinewood nematode (*Bursaphelenchus xylophilus*) by promoting plant antioxidative metabolism. Sci. Rep. 11 (1), 3781–3781. doi: 10.1038/s41598-021-83445-0 33580134 PMC7881030

[B42] Pang Y. Abeysinghe I. S. B. He J. He X. Huhman D. Mewan K. M. . (2013). Functional characterization of proanthocyanidin pathway enzymes from tea and their application for metabolic engineering. Plant Physiol. 161 (3), 1103–1116. doi: 10.1104/pp.112.212050 23288883 PMC3585583

[B43] Pang Y. Peel G. J. Wright E. Wang Z. Dixon R. A. (2007). Early steps in proanthocyanidin biosynthesis in the model legume *Medicago truncatula* . Plant Physiol. 145 (3), 601–615. doi: 10.1104/pp.107.107326 17885080 PMC2048810

[B44] Pfaffl M. W. Tichopad A. Prgomet C. Neuvians T. P. (2004). Determination of stable housekeeping genes, differentially regulated target genes and sample integrity: BestKeeper–excel-based tool using pair-wise correlations. Biotechnol. Lett. 26 (6), 509–515. doi: 10.1023/b:bile.0000019559.84305.47 15127793

[B45] Ren L. Wang M.-R. Wang Q.-C. (2021). ROS-induced oxidative stress in plant cryopreservation: occurrence and alleviation. Planta 254 (6), 124–124. doi: 10.1007/s00425-021-03784-0 34800184 PMC8605965

[B46] Shi J. Yu J. Pohorly J. E. Kakuda Y. (2003). Polyphenolics in grape seeds-biochemistry and functionality. J. Med. Food 6 (4), 291–299. doi: 10.1089/109662003772519831 14977436

[B47] Šoln K. Koce J. D. (2022). Oxidative stress in roots: Detection of lipid peroxidation and total antioxidative capacity. Methods Mol Biol 2447, 221–231. doi: 10.1007/978-1-0716-2079-3_18 35583785

[B48] Szankowski I. Flachowsky H. Li H. Halbwirth H. Treutter D. Regos I. . (2009). Shift in polyphenol profile and sublethal phenotype caused by silencing of anthocyanidin synthase in apple (*Malus* sp.). Planta 229 (3), 681–692. doi: 10.1007/s00425-008-0864-4 19066943

[B49] Trudgill D. L. (1991). Resistance to and tolerance of plant parasitic nematodes in plants. Annu. Rev. Phytopathol. 29 (1), 167–192. doi: 10.1146/annurev.py.29.090191.001123

[B50] Tsai I. J. Tanaka R. Kanzaki N. Akiba M. Yokoi T. Espada M. . (2016). Transcriptional and morphological changes in the transition from mycetophagous to phytophagous phase in the plant-parasitic nematode *Bursaphelenchus xylophilus* . Mol. Plant Pathol. 17 (1), 77–83. doi: 10.1111/mpp.12261 25831996 PMC6638504

[B51] Ullah C. Unsicker S. B. Fellenberg C. Constabel C. P. Schmidt A. Gershenzon J. . (2017). Flavan-3-ols are an effective chemical defense against rust infection. Plant Physiol. 175 (4), 1560–1578. doi: 10.1104/pp.17.00842 29070515 PMC5717727

[B52] Wang F. Chen Q. Zhang R. Li D. Ling Y. Song R. (2019). The anti-phytoalexin gene *Bx-cathepsin w* supports the survival of *Bursaphelenchus xylophilus* under *Pinus massoniana* phytoalexin stress. BMC Genomics 20 (1), 779. doi: 10.1186/s12864-019-6167-2 31655568 PMC6815438

[B53] Wang L. Jiang Y. Yuan L. Lu W. Yang L. Karim A. . (2013). Isolation and characterization of cDNAs encoding leucoanthocyanidin reductase and anthocyanidin reductase from *Populus trichocarpa* . PloS One 8 (5), e64664–e64664. doi: 10.1371/journal.pone.0064664 23741362 PMC3669385

[B54] Wei M. Chen Y. Zhang M. Yang J. Lu H. Zhang X. . (2020). Selection and validation of reference genes for the qRT-PCR assays of *Populus ussuriensis* gene expression under abiotic stresses and related ABA treatment. Forests 11, 476. doi: 10.3390/f11040476

[B55] Wen B. Mei Z. Zeng C. Liu S. (2017). metaX: a flexible and comprehensive software for processing metabolomics data. BMC Bioinf. 18 (1), 183. doi: 10.1186/s12859-017-1579-y PMC5361702 28327092

[B56] Wingfield M. J. (1982). Association of pine wood nematode with stressed trees in Minnesota, Iowa, and Wisconsin. Plant Dis. 66 (1), 934–937. doi: 10.1094/PD-66-934

[B57] Xin Y. Meng S. Ma B. He W. He N. (2020). Mulberry genes *MnANR* and *MnLAR* confer transgenic plants with resistance to *Botrytis cinerea* . Plant Sci. 296, 110473. doi: 10.1016/j.plantsci.2020.110473 32540003

[B58] Xu J. Z. Yeung S. Y. Chang Q. Huang Y. Chen Z. Y. (2004). Comparison of antioxidant activity and bioavailability of tea epicatechins with their epimers. Br. J. Nutr. 91 (6), 873–881. doi: 10.1079/bjn20041132 15182391

[B59] Xu L. Zhang J. Gao J. Chen X. Jiang C. Hao Y. (2012). Study on the disease resistance of candidate clones in *Pinus massoniana* to *Bursaphelenchus xylophilus* . China For. Sci. Technol. 26 (04), 27–30. doi: 10.3969/j.issn.1000-8101.2012.04.007

[B60] Yamada T. (2006). Biochemical responses in pines infected with *Bursaphelenchus xylophilus* . J. Japanese For. Soc. (Japan) 88 (5), 370–382. doi: 10.4005/jjfs.88.370

[B61] Yamada T. (2008). “Biochemical responses in pine trees affected by pine wilt disease,” in Pine wilt disease. Eds. Zhao B. G. Futai K. Sutherland J. R. Takeuchi Y. (Tokyo: Springer Japan), 223–234.

[B62] Yang B. Pan H. Tang J. Wang Y. Wang L. (2003). Pine wilt disease (Beijing, China: China Forestry Publishing House).

[B63] Ye J. (2019). Epidemic status of pine wilt disease in China and its prevention and control techniques and counter measures. Sci. Silvae Sinicae 55 (9), 10. doi: 10.11707/j.1001-7488.20190901

[B64] Yu H. Wu H. (2018). Discovery of new host plants and new insect vector of *Bursaphelenchus xylophilus* in liaoning province. For. Pest Dis. 37 (05), 61. doi: 10.3969/j.issn.1671-0886.2018.05.015

[B65] Yu D. Zhou L. Zhou W. Ding H. Wang Q. Wang Y. . (2011). Forest management in northeast China: history, problems, and challenges. Environ. Manage 48 (6), 1122–1135. doi: 10.1007/s00267-011-9633-4 21350964

[B66] Zhang Y. Lin X. (2019). Salicylic acid: biosynthesis, perception, and contributions to plant immunity. Curr. Opin. Plant Biol. 50, 29–36. doi: 10.1016/j.pbi.2019.02.004 30901692

[B67] Zhang M. Vervoort L. Moalin M. Mommers A. Douny C. den Hartog G. J. M. . (2018). The chemical reactivity of (-)-epicatechin quinone mainly resides in its b-ring. Free Radical Biol. Med. 124, 31–39. doi: 10.1016/j.freeradbiomed.2018.05.087 29859347

[B68] Zhao J. Han X. Shi J. (2017). Potential distribution of Bursaphelenchus xylophilus in China due to adaptation cold conditions. J. Biosafety 26 (3), 191–198. doi: 10.3969/j.issn.2095-1787.2017.03.003

[B69] Zhu G. Wang S. Huang Z. Zhang S. Liao Q. Zhang C. . (2018). Rewiring of the fruit metabolome in tomato breeding. Cell 172 (1), 249–261.e212. doi: 10.1016/j.cell.2017.12.019 29328914

