# Supplementary material for: Antioxidant processes involving epicatechin decreased symptoms of pine wilt disease
Source: Front Plant Sci. 2022 Dec 9;13:1015970. doi: 10.3389/fpls.2022.1015970 (PMC9780601; doi:10.3389/fpls.2022.1015970)
Supplement: Supplementary file 1 [file DataSheet_1.docx]

Supplementary Material

# Supplementary Figures and Tables

## Supplementary Figures


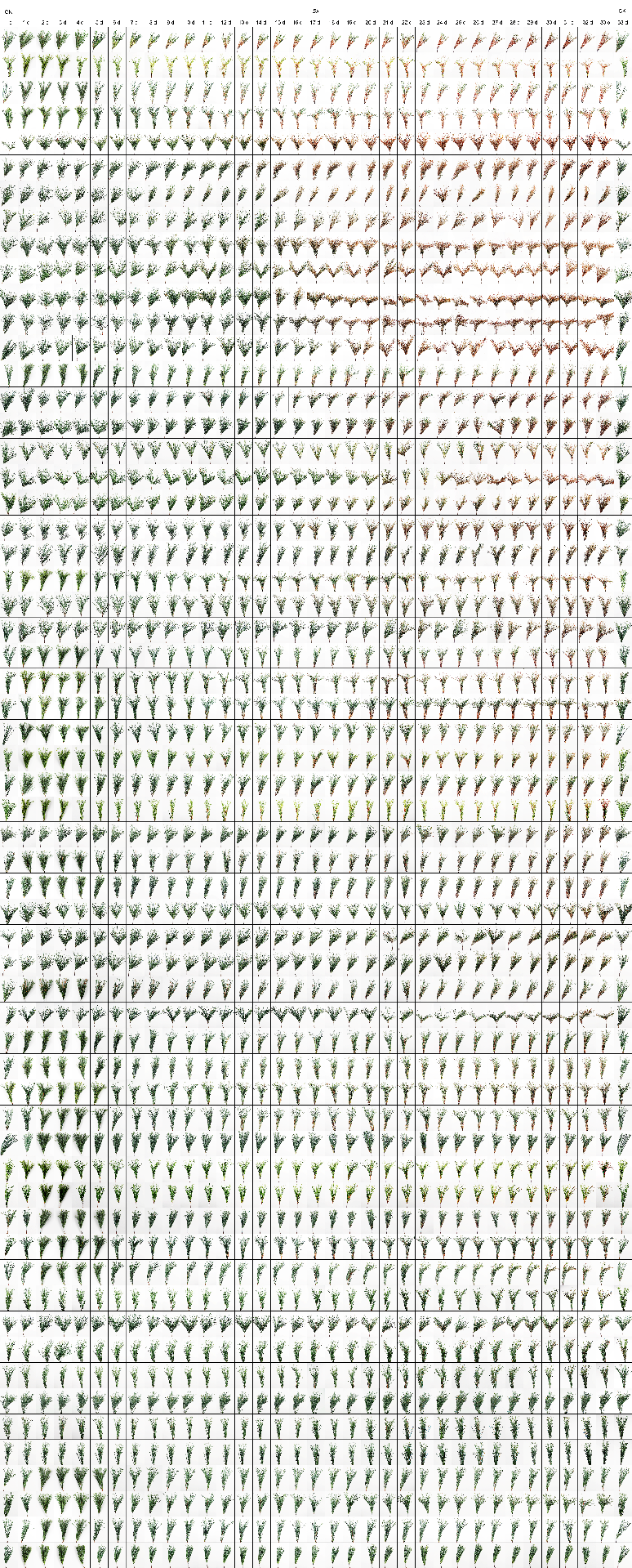


**Supplementary Figure 1.** Symptom of 60 *P. koraiensis* individuals inoculated with FS*Bx*

**
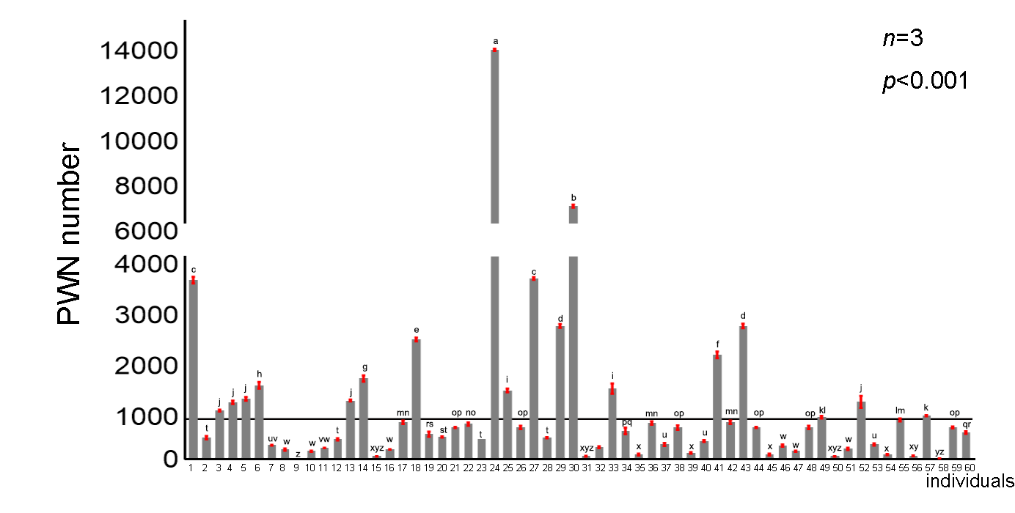
**

**Supplementary Figure 2.** Number of PWNs isolated from branches of 60 *P. koraiensis* individuals

Data in **Supplementary Figure 2** were analyzed by one-way ANOVA followed by Tukey’s posthoc test, with different letters indicating statistically significant differences at 95% confidence. The data in the figures are means ± SE (*n*=3). Letter a: the maximum average number marked with the letter a. Letter b: The maximum average is compared with the following averages. Where the difference is not significant, the letter a is marked until a significant difference is marked with the letter b. Letter labeling followed by analogy. Where there is an identically marked letter, the difference is not significant; where there are different marked letters, the difference is significant.


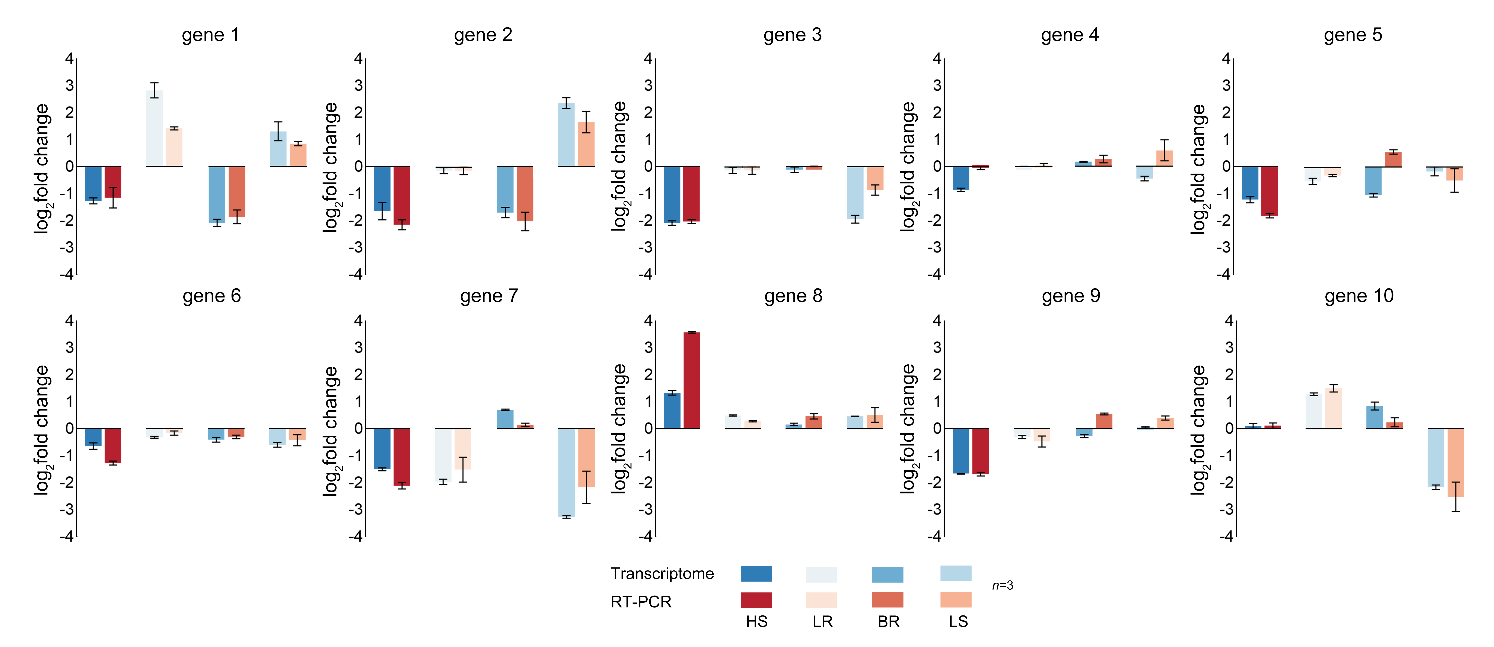


**Supplementary Figure 3.** RT-PCR verification of DEGs in the transcriptome


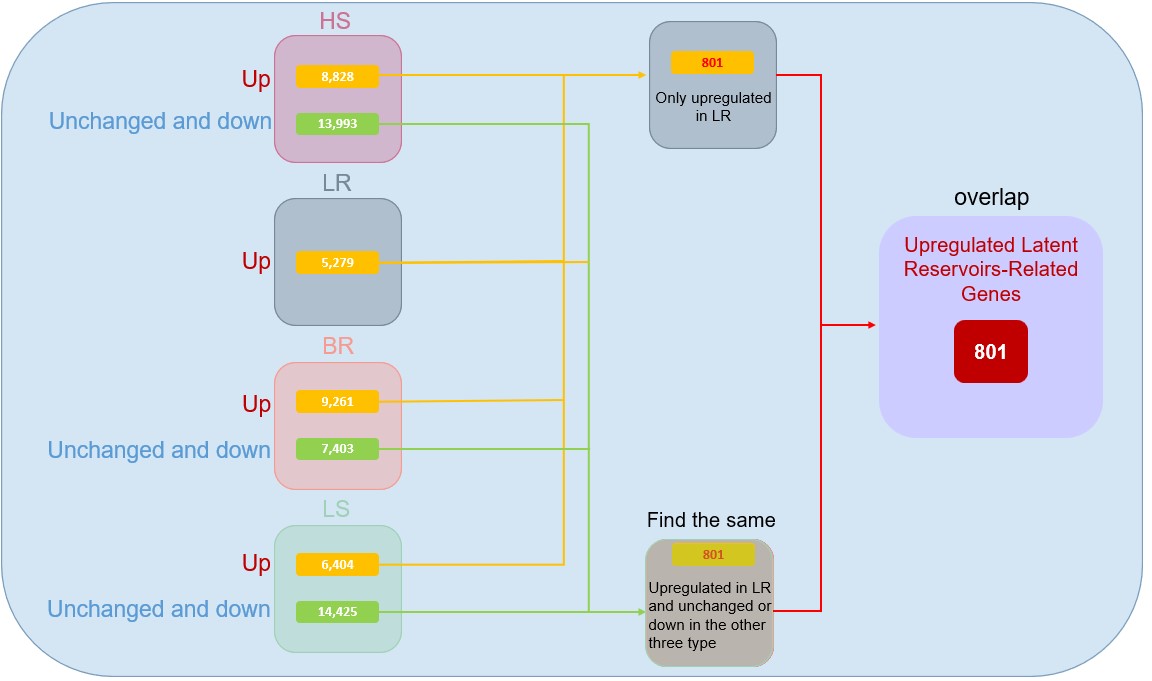


**Supplementary Figure 4.** Identification of upregulated Latent Reservoirs-related genes

**
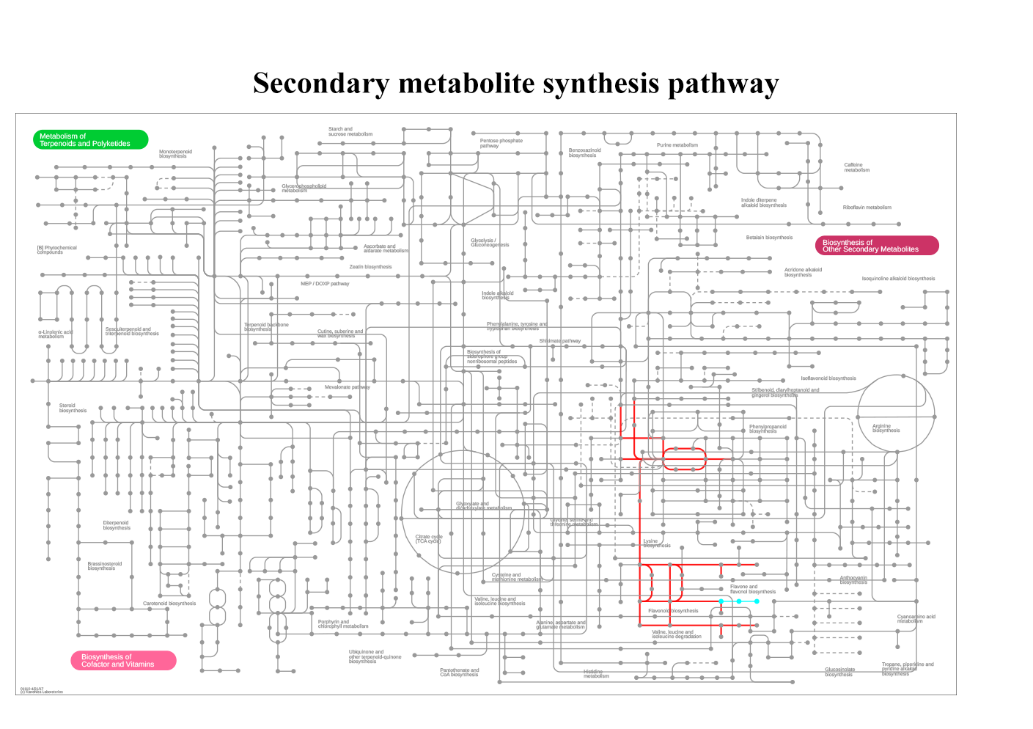
**

**Supplementary Figure 5.** Secondary metabolite synthesis pathway


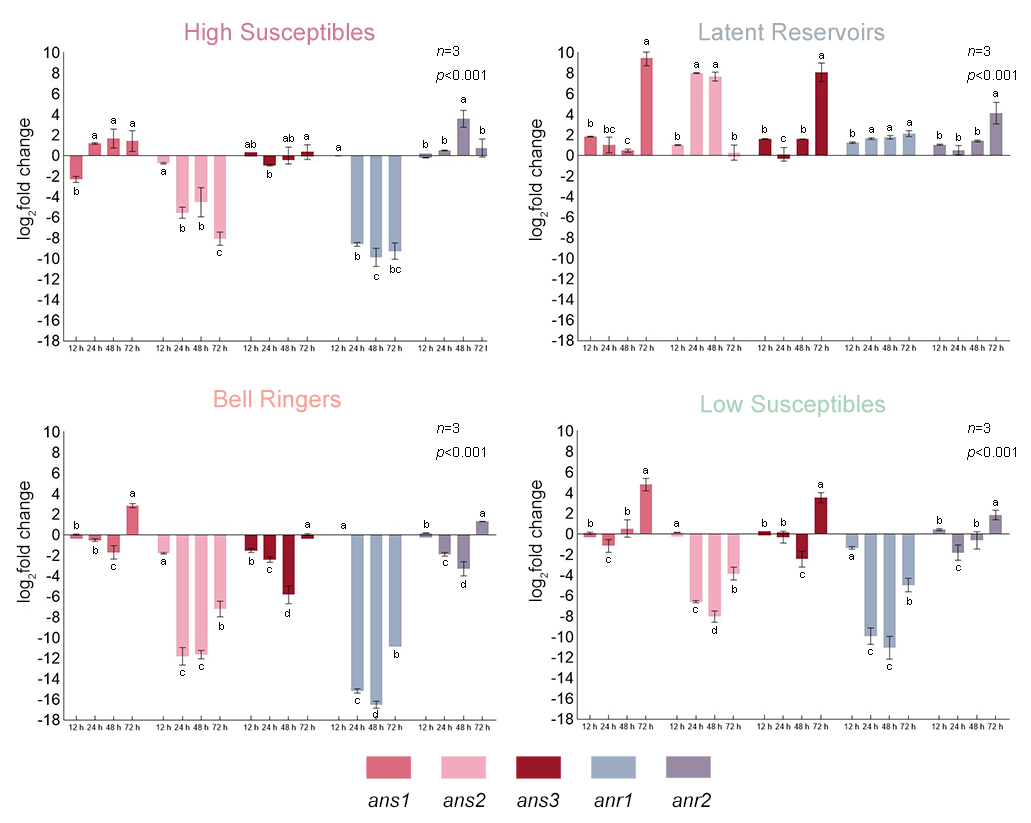


**Supplementary Figure 6.** Expression levels of 3 *ans* and 2 *anr* genes in 4 types of *P. koraiensis* at 4 times

Data in **Supplementary Figure 6** were analyzed by one-way ANOVA followed by Tukey’s posthoc test, with different letters indicating statistically significant differences at 95% confidence. The data in the figures are means ± SE (*n*=3). Letter a: the maximum average number marked with the letter a. Letter b: The maximum average is compared with the following averages. Where the difference is not significant, the letter a is marked until a significant difference is marked with the letter b. Letter labeling followed by analogy. Where there is an identically marked letter, the difference is not significant; where there are different marked letters, the difference is significant.


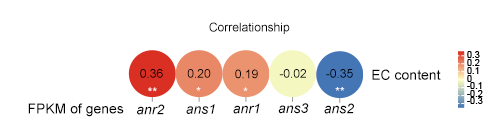


**Supplementary Figure 7.** Correlation analysis between EC content and 5 genes FPKM

The *p*-value is the significant value, marked with *. Labeling rules are: *p* > = 0.05, no label, 0.01 < *p* < 0.05, label: *, 0.001 < *p* < 0.01, label: **, *p* < = 0.001, label ***.


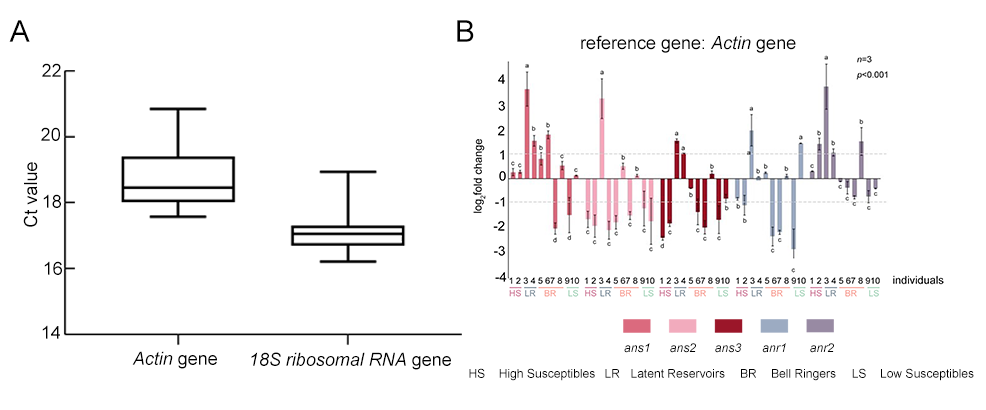


**Supplementary Figure 8.** Screening of Reference Genes

**A:** The Cts of two reference genes in 20 different samples. **B:** Expression levels of 3 *ans* and 2 *anr* genes with *Actin* as reference gene in 10 *P. koraiensis* individuals at 12 hpi

Data in **Supplementary Figure 8B** were analyzed by one-way ANOVA followed by Tukey’s posthoc test, with different letters indicating statistically significant differences at 95% confidence. The data in the figures are means ± SE (*n*=3). Letter a: the maximum average number marked with the letter a. Letter b: The maximum average is compared with the following averages. Where the difference is not significant, the letter a is marked until a significant difference is marked with the letter b. Letter labeling followed by analogy. Where there is an identically marked letter, the difference is not significant; where there are different marked letters, the difference is significant.


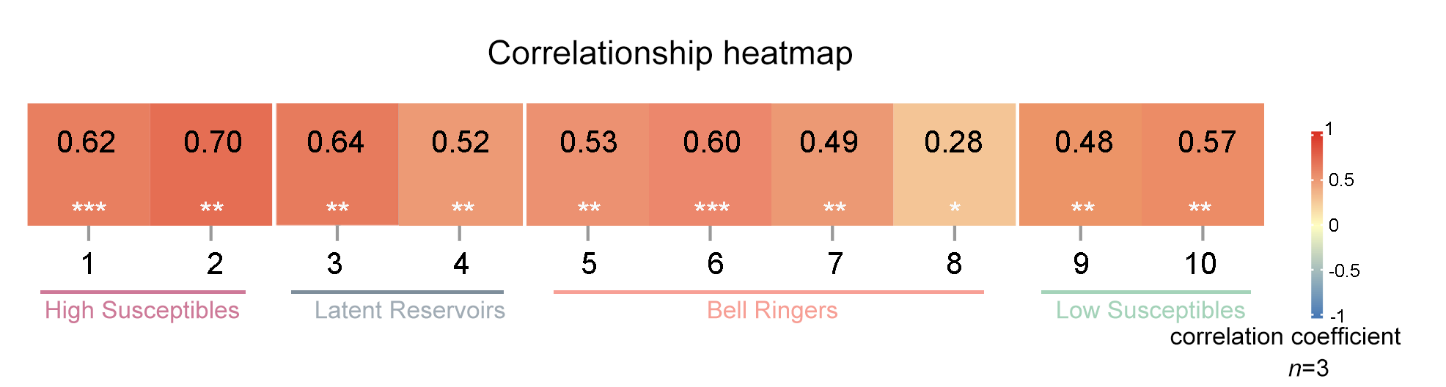


**Supplementary Figure 9.** Correlation analysis of 5 genes in the same type of *P. koraiensis* individuals

The *p*-value is the significant value, marked with *. Labeling rules are: *p* > = 0.05, no label, 0.01 < *p* < 0.05, label: *, 0.001 < *p* < 0.01, label: **, *p* < = 0.001, label ***.


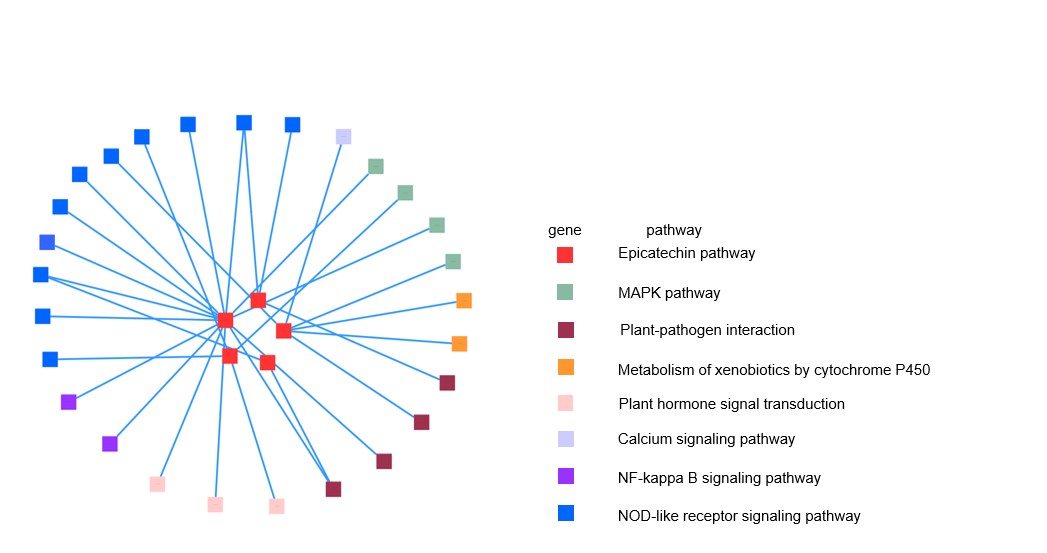


**Supplementary Figure 10.** Network associated with EC

## Supplementary Tables

**Supplementary Table 1** RT–PCR Primers used in this experiment

| Gene | Forward primer | Reverse primer |
| --- | --- | --- |
| *ans1* | ATTCGAGTGGCTAATGGCG | CAGAATGGGGTGCCAATCC |
| *ans2* | CGTTGTCCACAACCAGAGC | GTCACCAATGTGGACGATGA |
| *ans3* | CTGTCCACAACCGGAACTC | AATATCTCGACTTGGTCGCC |
| *anr1* | TCCACCTGGCGCTCTC | CGAGGTGTTGATGGGACAA |
| *anr2* | CAAGGCCCACATCTTCTTGA | GACAGGTTTGAGATCGGTGG |
| *18S* | ACCATAACACTCGCGACAAA | GTAGTATGTGGAAGGGTGCG |
| *Actin* | TCTGGTCCTTCCATCGTCC | CTTAGCACTATTGCCATCATCT |
| gene 1 | CGTGGCTCAAGTCGAGTATT | CGGGCTCGAAAATGGATGTA |
| gene 2 | AGAGGAAGCTATGGGGGTTT | TCCCATAGTAAGCCAACCCA |
| gene 3 | GCATCTGCTCCCTCATATCC | TTAATGTCTCACTCTCCTCGG |
| gene 4 | TGAATGGACCAATTCCAAATGT | AATTGTTGAGGAAGCTCGC |
| gene 5 | ATTGACGCTGGATGAGTGC | GTCCTCCACGTCCAAGACTC |
| gene 6 | ACCTGGAAGAGTACCAGTGA | CATCATCATGTCCCTTGGCA |
| gene 7 | ACTCTAAGAAGAAGAGCCGGA | CACAGTTGCTATTGGAGCATC |
| gene 8 | CAATCATCCATGGCCTGCTA | TCTTCTGGAGTGGATGCTAC |
| gene 9 | CAATCCGACTGTGTACGAGC | TTGATGTGCGAGGCCATTAC |
| gene 10 | ATAACATCACCCTGTTCCGTC | GGGAGGAAGAGATCACTGGT |

**Supplementary Table 2** Disease infection index of 60 *P. koraiensis* individuals

| type | proportion of green needles | proportion of brown needles | DI | scale |
| --- | --- | --- | --- | --- |
| High Susceptibles | 0 | 1 | 1 | IV |
|  | 0 | 1 | 1 | IV |
|  | 0 | 1 | 1 | IV |
|  | 0 | 1 | 1 | IV |
|  | 0 | 1 | 1 | IV |
|  | 0.05±0.006 | 0.95±0.006 | 0.95±0.006 | IV |
|  | 0.051±0.011 | 0.949±0.011 | 0.949±0.011 | IV |
|  | 0.092±0.005 | 0.908±0.005 | 0.908±0.005 | IV |
|  | 0.146±0.004 | 0.854±0.004 | 0.854±0.004 | IV |
|  | 0.169 | 0.831 | 0.831 | IV |
|  | 0.178±0.0011 | 0.822±0.0011 | 0.822±0.0011 | IV |
|  | 0.183±0.0049 | 0.817±0.0049 | 0.817±0.0049 | IV |
|  | 0.211±0.0013 | 0.789±0.0013 | 0.789±0.0013 | IV |
|  | 0.377±0.012 | 0.623±0.012 | 0.623±0.012 | III |
|  | 0.523±0.02 | 0.477±0.02 | 0.477±0.02 | II |
|  | 0.565±0.044 | 0.435±0.044 | 0.435±0.044 | II |
|  | 0.845±0.0295 | 0.155±0.0295 | 0.155±0.0295 | I |
| Latent Reservoirs | 0.92±0.04 | 0.08±0.04 | 0.08±0.04 | 0 |
|  | 0.96±0.04 | 0.04±0.04 | 0.08±0.08 | 0 |
|  | 0.98±0.0087 | 0.02±0.0087 | 0.04±0.0087 | 0 |
| type | proportion of green needles | proportion of brown needles | DI | scale |
| Bell Ringers | 0 | 1 | 1 | IV |
|  | 0.022±0.003 | 0.978±0.003 | 0.978±0.003 | IV |
|  | 0.032±0.004 | 0.968±0.004 | 0.968±0.004 | IV |
|  | 0.036±0.006 | 0.964±0.006 | 0.964±0.006 | IV |
|  | 0.041±0.0015 | 0.959±0.0015 | 0.959±0.0015 | IV |
|  | 0.042±0.002 | 0.958±0.002 | 0.958±0.002 | IV |
|  | 0.046±0.0018 | 0.954±0.0018 | 0.954±0.0018 | IV |
|  | 0.058±0.0046 | 0.942±0.0046 | 0.942±0.0046 | IV |
|  | 0.063±0.0019 | 0.937±0.0019 | 0.937±0.0019 | IV |
|  | 0.083±0.0026 | 0.917±0.0026 | 0.917±0.0026 | IV |
|  | 0.113±0.007 | 0.887±0.007 | 0.887±0.007 | IV |
|  | 0.119±0.012 | 0.881±0.012 | 0.881±0.012 | IV |
|  | 0.123±0.0009 | 0.877±0.0009 | 0.877±0.0009 | IV |
|  | 0.129±0.005 | 0.871±0.005 | 0.871±0.005 | IV |
|  | 0.14±0.007 | 0.86±0.007 | 0.86±0.007 | IV |
|  | 0.173±0.001 | 0.827±0.001 | 0.827±0.001 | IV |
|  | 0.18±0.0062 | 0.82±0.0062 | 0.82±0.0062 | IV |
|  | 0.304±0.048 | 0.696±0.048 | 0.696±0.048 | III |
|  | 0.341±0.0235 | 0.659±0.0235 | 0.659±0.0235 | III |
| Bell Ringers | 0.422±0.124 | 0.578±0.124 | 0.578±0.124 | III |
|  | 0.427±0.017 | 0.573±0.017 | 0.573±0.017 | III |
|  | 0.486±0.0019 | 0.514±0.0019 | 0.514±0.0019 | III |
|  | 0.486 | 0.514 | 0.514 | III |
|  | 0.492±0.0044 | 0.508±0.0044 | 0.508±0.0044 | III |
|  | 0.498±0.014 | 0.502±0.014 | 0.502±0.014 | III |
|  | 0.511±0.006 | 0.489±0.006 | 0.489±0.006 | II |
|  | 0.545±0.01 | 0.455±0.01 | 0.455±0.01 | II |
|  | 0.586±0.037 | 0.414±0.037 | 0.414±0.037 | II |
|  | 0.613±0.083 | 0.387±0.083 | 0.387±0.083 | II |
|  | 0.671±0.018 | 0.329±0.018 | 0.329±0.018 | II |
|  | 0.735±0.033 | 0.265±0.033 | 0.265±0.033 | II |
|  | 0.811±0.034 | 0.189±0.034 | 0.189±0.034 | I |
|  | 0.856±0.0217 | 0.144±0.0217 | 0.144±0.0217 | I |
|  | 0.892±0.02 | 0.108±0.02 | 0.108±0.02 | I |
| Low Susceptibles | 0.96±0.008 | 0.04±0.008 | 0.04±0.008 | 0 |
|  | 0.974±0.016 | 0.026±0.016 | 0.026±0.016 | 0 |
|  | 0.99±0.005 | 0.01±0.005 | 0.01±0.005 | 0 |
|  | 1 | 0 | 0 | 0 |
|  | 1 | 0 | 0 | 0 |
|  | 1 | 0 | 0 | 0 |

**Supplementary Table 3** Sixty *P. koraiensis* individuals classification according to the number of isolated PWNs

| Type | individuals number |
| --- | --- |
| individuals with relatively more PWNs | 20 |
| individuals with fewer PWNs | 40 |

**Supplementary Table 4** Number of individuals of 4 Types of *P. koraiensis*

| Type | individuals number |
| --- | --- |
| High Susceptibles | 17 |
| Latent Reservoirs | 3 |
| Bell Ringers | 34 |
| Low Susceptibles | 6 |

**Supplementary Table 5** Quality statistics of filtered reads

| Sample | Total Raw Reads (M) | Total Clean Reads (M) | Total Clean Bases(Gb) | Clean Reads Q20(%) | Clean Reads Q30(%) | Clean Reads Ratio(%) |
| --- | --- | --- | --- | --- | --- | --- |
| Treatment 1 | 43.82 | 42.59 | 6.39 | 96.28 | 90.82 | 97.18 |
| CK1 | 43.82 | 42.82 | 6.42 | 96.41 | 91.13 | 97.72 |
| Treatment 2 | 43.82 | 42.36 | 6.35 | 96.58 | 91.54 | 96.68 |
| CK2 | 43.82 | 42.49 | 6.37 | 96.48 | 91.33 | 96.96 |
| Treatment 1 | 43.82 | 42.66 | 6.4 | 96.63 | 91.65 | 97.34 |
| CK3 | 43.82 | 42.73 | 6.41 | 96.59 | 91.55 | 97.52 |
| Treatment 1 | 43.82 | 42.69 | 6.4 | 96.52 | 91.37 | 97.43 |
| CK4 | 43.82 | 42.59 | 6.39 | 96.66 | 91.72 | 97.19 |

**Supplementary Table 6** Identification of differentially expressed genes

| Sample | Non DEG | DEGs | Up-related | Down-related |
| --- | --- | --- | --- | --- |
| High Susceptibles |  | 21,632 | 8,828 | 12,804 |
| Latent Reservoirs |  | 8,472 | 5,279 | 3,193 |
| Bell Ringers |  | 15,303 | 9,261 | 6,042 |
| Low Susceptibles |  | 17,800 | 6,404 | 11,396 |

**Supplementary Table 7** KEGG enrichment result for genes in the turquoise module (top 20 pathways)

| KEGG_A_class | KEGG_B_class | Pathway | count (268) | Pathway ID |
| --- | --- | --- | --- | --- |
| Genetic Information Processing | Translation | Ribosome | 69 | ko03010 |
| Metabolism | Biosynthesis of other secondary metabolites | Flavonoid biosynthesis | 25 | ko00941 |
| Metabolism | Lipid metabolism | Synthesis and degradation of ketone bodies | 5 | ko00072 |
| Metabolism | Metabolism of other amino acids | Cyanoamino acid metabolism | 6 | ko00460 |
| KEGG_A_class | KEGG_B_class | Pathway | count (268) | Pathway ID |
| Metabolism | Carbohydrate metabolism | Butanoate metabolism | 7 | ko00650 |
| Organismal Systems | Environmental adaptation | Circadian rhythm - plant | 9 | ko04712 |
| Genetic Information Processing | Translation | RNA transport | 16 | ko03013 |
| Cellular Processes | Transport and catabolism | Endocytosis | 8 | ko04144 |
| Metabolism | Metabolism of terpenoids and polyketides | Carotenoid biosynthesis | 6 | ko00906 |
| Metabolism | Energy metabolism | Nitrogen metabolism | 3 | ko00910 |
| Genetic Information Processing | Folding, sorting and degradation | Protein processing in endoplasmic reticulum | 11 | ko04141 |
| Metabolism | Metabolism of cofactors and vitamins | Porphyrin and chlorophyll metabolism | 4 | ko00860 |
| Metabolism | Glycan biosynthesis and metabolism | Other types of O-glycan biosynthesis | 2 | ko00514 |
| Genetic Information Processing | Folding, sorting and degradation | Protein export | 2 | ko03060 |
| Genetic Information Processing | Transcription | Spliceosome | 14 | ko03040 |
| Metabolism | Glycan biosynthesis and metabolism | Glycosaminoglycan degradation | 1 | ko00531 |
| Metabolism | Carbohydrate metabolism | C5-Branched dibasic acid metabolism | 1 | ko00660 |
| Metabolism | Nucleotide metabolism | Pyrimidine metabolism | 3 | ko00240 |
| Metabolism | Metabolism of terpenoids and polyketides | Terpenoid backbone biosynthesis | 7 | ko00900 |
| Metabolism | Amino acid metabolism | Lysine biosynthesis | 1 | ko00300 |

**Supplementary Table 8** Changes of H_2_O_2_ content in Treatment group and CK group of 4 Types of *P. koraiensis*

| Type | CK1 | CK2 | CK3 | Treatment1 | Treatment2 | Treatment3 | ratio | *P* value |
| --- | --- | --- | --- | --- | --- | --- | --- | --- |
| HS | 82.60 | 81.36 | 80.69 | 90.04 | 91.12 | 90.37 | 1.11 | 0.000592 |
| LR | 102.36 | 103.69 | 103.36 | 108.50 | 108.87 | 107.49 | 1.05 | 0.000857 |
| BR | 96.64 | 95.38 | 94.29 | 114.03 | 108.73 | 109.38 | 1.16 | 0.005477 |
| LS | 78.86 | 79.63 | 76.89 | 82.80 | 82.82 | 83.81 | 0.29 | 0.01767 |

**Supplementary Table 9** Changes of EC content in Treatment group and CK group of 4 Types of *P. koraiensis*

| Type | CK1 | CK2 | CK3 | Treatment1 | Treatment2 | Treatment3 | ratio | *P* value |
| --- | --- | --- | --- | --- | --- | --- | --- | --- |
| HS | 17856142 | 16563020 | 15698030 | 3749790 | 3809495 | 4395448 | 0.24 | 0.001028 |
| LR | 25485869 | 21698040 | 26980520 | 9939489 | 8245255 | 9173377 | 0.37 | 0.006019 |
| BR | 12798421 | 10026892 | 14506938 | 2047747 | 1403765 | 3481665 | 0.18 | 0.007043 |
| LS | 12442745 | 14569820 | 12639506 | 3235114 | 4516644 | 3791852 | 0.29 | 0.001036 |

**Supplementary Table 10** Expression Stability of Candidate Reference Genes Calculated by BestKeeper

| Rank | Gene name | SD | CV |
| --- | --- | --- | --- |
| 1 | *18S ribosomal* *RNA* | 0.90 | 4.82% |
| 2 | *Actin* | 0.63 | 3.69% |

# Supplementary Method

**2.1 Relative Quantification**

The Cts of 3 *ans* and 2 *anr* genes and reference gene in the treatment group and CK group were obtained respectively. The relative quantification method was used to calculate the data. The RT-PCR results were normalized (log_2_[fold-change]) by reference gene to those of constitutively expressed genes. The calculation formula of fold-change was as follows:

∆Ct(Treatment)=Ct(gene_Treatment)-Ct(reference gene_Treatment)

∆Ct(CK)=Ct(gene_CK)-Ct(reference gene_CK)

∆∆Ct=∆Ct(Treatment)-∆Ct(CK)

fold-change=2^(-∆∆Ct)

**2.2 Construction of RNA Libraries**

1) Total RNA was processed by mRNA enrichment or rRNA removal. mRNA enrichment: mRNA with polyA tail was enriched by magnetic beads with OligodT; rRNA removal: The rRNA is hybridized with a DNA probe, RNaseH selectively digests the DNA/RNA hybrid strand, and DNaseI digests the DNA probe to obtain the desired RNA after purification.

2) The obtained RNA was fragmented by interrupting the buffer, and the random N6 primers were reverse transcribed, and then the cDNA double-stranded was synthesized to form double-stranded DNA.

3) The end of the synthesized double-stranded DNA was flattened and the 5 ' end was phosphorylated. The 3 ' end formed a sticky end that highlighted an ' A ', and then connected to a 3 ' end with a bulged ' T '.

4) The ligation product was amplified by PCR with specific primers.

5) The PCR product was thermally denatured into a single strand, and a single-strand circular DNA library was obtained by cyclization of the single-strand DNA with a bridge primer.

**2.3 Extraction and Analysis of Metabolites**

Fifty mg samples were weighed and placed in a 1.5 mL Eppendorf tube, and 800 μL extract (methanol: water = 7:3, v/v, -20℃ precooling ) and 20 μL internal standard was added. Two small steel balls were added and placed in a tissue grinding instrument for grinding (50 Hz, 5 min). After ultrasonic treatment at 4°C for 30 min, the samples were placed in a refrigerator at -20℃ for 1 h. 4°C, 14000 rpm for 15 min. After centrifugation, 600 μL of supernatant was taken and filtered through a 0.22 μm filter membrane. The filtered samples were placed in a loading bottle for LC-MS analysis. Each sample was mixed into 20 μL quality control (QC) samples to evaluate the repeatability and stability of the LC-MS analysis process.

In this study, Waters 2D UPLC (waters, USA) coupled with Q Exactive high-resolution mass spectrometer (Thermo Fisher Scientific, USA) was used to separate and detect metabolites. The chromatographic column used was Hypersil GOLD aQ chromatographic column (100*2.1 mm, 1.9 μm, Thermo Fisher Scientific, USA). The mobile phase consisted of the aqueous solution containing 0.1% formic acid (solution A) and 100 % acetonitrile containing 0.1% formic acid (solution B). Q Exactive mass spectrometer (Thermo Fisher Scientific, USA) was used to collect primary and secondary mass spectrometry data. The mass-to-nuclear ratio range of mass spectrometry was 150-1500, the first-order resolution was 70,000, the AGC was 1e6, and the maximum injection time (IT) was 100 ms.

**2.4** **The *P*-value calculation method in KEGG enrichment analysis**

The calculation formula of the *P*-value is as follows：

$$P=1-\sum_{i=0}^{m-1} \frac{\left( \begin{matrix} M \\ i \end{matrix} \right)\left( \begin{matrix} N-M \\ n-i \end{matrix} \right)}{\left( \begin{matrix} N \\ n \end{matrix} \right)}$$

Among them, *N* is the number of all genes, *n* is the number of differentially expressed genes, *M* is the number of KEGG pathways in all genes, and *i* is the number of annotated pathways in differentially expressed genes. After the calculated *P*-value is corrected by FDR, the corrected *P*-value ≤ 0.05 is used as the threshold. The pathway that meets this condition is defined as a pathway that is significantly enriched in differentially expressed genes.
